# Cellular phosphatases facilitate combinatorial processing of receptor-activated signals

**DOI:** 10.1186/1756-0500-1-81

**Published:** 2008-09-17

**Authors:** Dhiraj Kumar, Raina Dua, Ravichandran Srikanth, Shilpi Jayaswal, Zaved Siddiqui, Kanury VS Rao

**Affiliations:** 1 Immunology Group, International Centre for Genetic Engineering and Biotechnology, Aruna Asaf Ali Marg, New Delhi, 110067, India

## Abstract

**Background:**

Although reciprocal regulation of protein phosphorylation represents a key aspect of signal transduction, a larger perspective on how these various interactions integrate to contribute towards signal processing is presently unclear. For example, a key unanswered question is that of how phosphatase-mediated regulation of phosphorylation at the individual nodes of the signaling network translates into modulation of the net signal output and, thereby, the cellular phenotypic response.

**Results:**

To address the above question we, in the present study, examined the dynamics of signaling from the B cell antigen receptor (BCR) under conditions where individual cellular phosphatases were selectively depleted by siRNA. Results from such experiments revealed a highly enmeshed structure for the signaling network where each signaling node was linked to multiple phosphatases on the one hand, and each phosphatase to several nodes on the other. This resulted in a configuration where individual signaling intermediates could be influenced by a spectrum of regulatory phosphatases, but with the composition of the spectrum differing from one intermediate to another. Consequently, each node differentially experienced perturbations in phosphatase activity, yielding a unique fingerprint of nodal signals characteristic to that perturbation. This heterogeneity in nodal experiences, to a given perturbation, led to combinatorial manipulation of the corresponding signaling axes for the downstream transcription factors.

**Conclusion:**

Our cumulative results reveal that it is the tight integration of phosphatases into the signaling network that provides the plasticity by which perturbation-specific information can be transmitted in the form of a multivariate output to the downstream transcription factor network. This output in turn specifies a context-defined response, when translated into the resulting gene expression profile.

## Background

Reciprocal regulation of protein phosphorylation by kinases and phosphatases represents a key aspect of signal transduction [1-6]. Although information on the role of phosphatases in regulating individual signaling modules continues to accumulate, a larger perspective on how these various interactions integrate to contribute towards signal processing is lacking [7-9]. To explore this we examined the dynamics of signaling from the B cell antigen receptor (BCR) under conditions where individual cellular phosphatases were selectively depleted by siRNA. We found that each phosphatase exhibited an extended sphere of influence where the rate, amplitude and duration of the signal at multiple nodes could be simultaneously affected. Thus, any perturbation in phosphatase activity was propagated in an unequal fashion across the network, thereby producing its own unique fingerprint in terms of nodal contribution to the net signal output. It was this property that ensured that the effector output of the signaling network could be manipulated in a combinatorial manner.

## Findings

### Phosphatase-mediated regulation of BCR signaling

**Figure 1:**
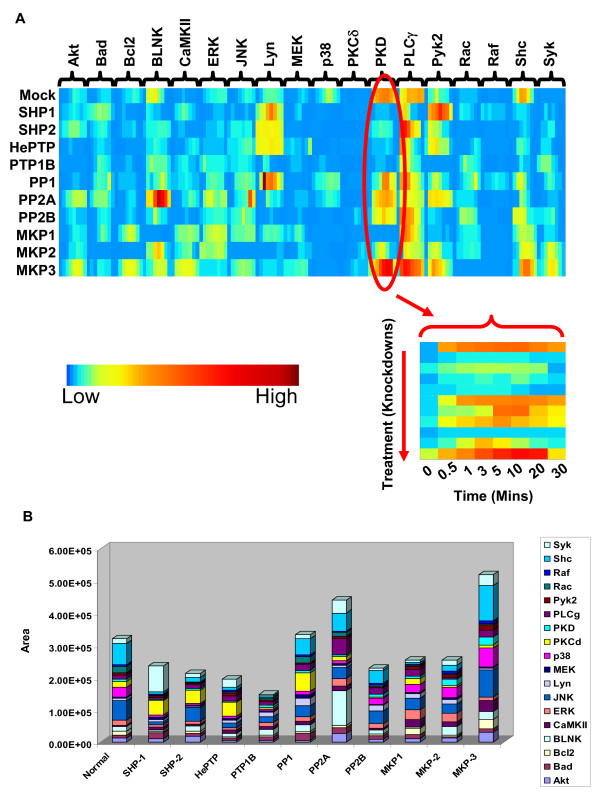
**Influence of Phosphatase knockdowns on BCR-dependent activation of signaling intermediates**. Panel 'A' depicts the kinetics of phosphorylation of select intermediates in the BCR signaling pathway. Normalized phosphorylation profiles are represented here as a heat map with rows showing various siRNA knockdowns and columns showing different signaling intermediates whose phosphorylation were measured. The blown out region shows such profiles of PKD under all the knockdown conditions. The color bar shows relative magnitude of phosphorylation at individual time points. Panel 'B' shows cumulative area under curve calculated for activation profile of all the signaling molecules under individual knockdown condition (see text). Total area under activation curve as well as individual contribution of signaling intermediates varied across the siRNA knockdown conditions. The height of each bar gives the net strength of BCR signal generated (see text) following transfection of cells either with Mock, or individual phosphatase-specific siRNA. The colored regions within each bar identify the signaling intermediates examined, and the area of spread represents their proportional contribution to the net signal strength.

Murinr B lymphoma, A20, cells were first individually depleted of one of a set of ten selected phosphatases siRNA. The extent of depletion varied 65% to 90% at the protein level (Additional file 1). Subsequently, these cells were stimulated with anti-IgG, and the time-dependent phosphorylation of a select panel of eighteen signaling intermediates was monitored[10]. Figure 1A summarizes the results obtained (see Additional files 2 and 3) in the form of a heat map. It is evident that silencing of any given phosphatase led to distinct effects on each of the signaling intermediates examined (Fig. 1A). However, the phosphatases involved and the extent of their effects differed between the intermediates (Fig. 1A). Conversely, each signaling molecule also displayed sensitivity to a broad range of phosphatases, although the effect varied depending upon which phosphatase was inhibited. For instance, the amplitude of BLNK phosphorylation was enhanced following PP2A-silencing, while it was attenuated either when PP1, SHP-1, HePTP, or MKP1 was suppressed (Fig. 1A). Thus, phosphatases appear to be intimately involved in shaping the phosphorylation profile of the various, BCR-dependent, signaling intermediates.

### Phosphatases modulate the signal output

We next determined the area under the phosphorylation curve (AUC) obtained for each intermediate, for each of the various conditions of perturbation. Although a gross approximation, we took this value to represent signal intensity at that particular node, under that specific perturbation condition. To estimate total flux of signal generated, the AUCs of each of the nodes under individual conditions were then summed up. Figure 1B reveals that cellular phosphatases influence the cumulative strength of receptor-dependent signal generated. Further, significant effects were also observed at the level of signal composition (Fig. 1B). Thus, contributions from Shc and JNK were substantially reduced in cells depleted either of SHP-1, HePTP, or PTP1B. In contrast, the effect was restricted to Shc in cells expressing reduced levels of SHP-2, or MKP1 (Fig. 1B). Such phosphatase-dependent variations in signal intensity were observed for all individual signaling intermediates examined, resulting in unique patterns of proportional contributions from the individual components to the signal strength. Thus, cellular phosphatases individually exert weighted effects on the signaling networks.

**Figure 2:**
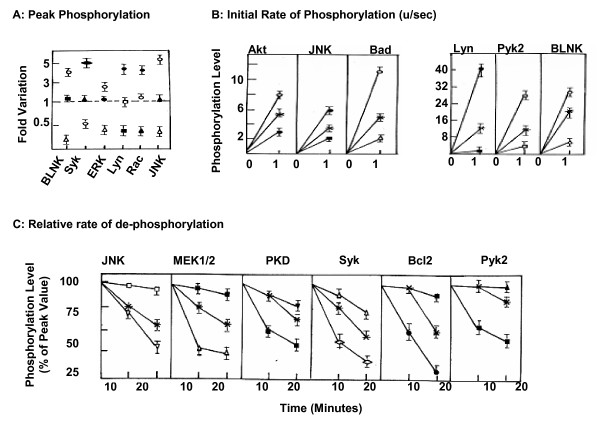
**Phosphatase-dependent regulation provides for the generation of signal plasticity**. Panels A, B and C depict the effect of silencing of either PP1 (●), PP2A (○), MKP3 (◆), MKP1 (□), MKP2 (■), SHP2 (∇), HePTP (▲), PP2B (▼), SHP1 (△) or PTP1B (◇) on peak phosphorylation levels, initial phosphorylation rate and rate of de-phosphorylation respectively on representative examples, as compared to that obtained for cells treated with non-silencing siRNA (dashed line in panel A, or * in panels B and C).

Figure 2 compares three different aspects of the phosphorylation curves obtained in Figure 1. These are; the peak phosphorylation level, the initial rate of activation (upto 1 min.), and the rate of subsequent dephosphorylation (decay rate) for the individual molecules. It is evident that all these three parameters displayed differential sensitivity to phosphatase depletion. Typical examples for each parameter are shown in Figure 2A, B and 2C. That both positive and negative effects can be seen in each case highlights the multiplicity of mechanisms that seem to be involved in the phosphatase-mediated regulation of BCR-dependent signaling.

### Activation-induced protein phosphorylation represents dynamic shifts in the kinase-phosphatase equilibrium

We next selected four target proteins that were stably phosphorylated – upon cell stimulation – to yield a plateau phase that was sustained over an extended period of time (i.e. Lyn, ERK, PLCγ, and JNK). Our aim was to ascertain whether this plateau phase truly described a stably phosphorylated state, or, if it simply identified an alteration in turnover between the phosphorylated and the dephosphorylated states of the protein.

**Figure 3:**
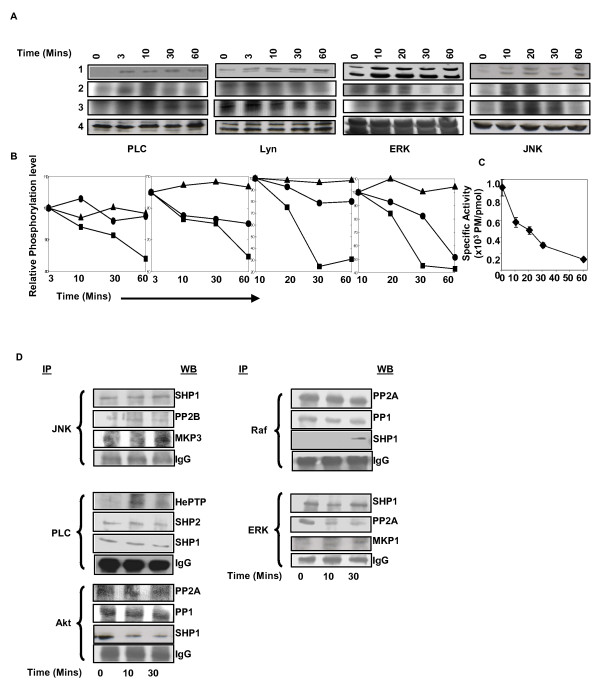
**The kinase-phosphatase dynamic equilibrium determines the activity profile of signaling intermediates**. Panel A, shows the results of a pulse-chase experiment (see text) where the indicated intermediates were immunoprecipitated and their phosphorylation status monitored either by Western blot (with phospho-specific antibodies, lane 1), or by autoradiography (lane 2). Lane 3 shows the results of a parallel experiment where phosphatase inhibitor (Sodium vanadate) was also included during the chase period. Lane 4 compares the amount of the parent protein present in each group as detected by Western blot using antibodies specific for the individual proteins. Panel B provides a graphical depiction of the relative stability of the phosphorylated forms of each of these intermediates after they reach the peak, when detected either by Western blot (left hand side), or by autoradiography (middle). The profile obtained in parallel groups that included phosphatase inhibitor is also shown (right hand side). Intensity values obtained for the data shown in panel A are plotted here, and values are the mean (± S.D.) of two independent experiments. The rate of turnover of the ^32^P-labelled γ phosphate of ATP, in these cells, is shown in panel C. Panel D shows the results of co-immunoprecipitation experiments where the indicated signaling intermediates (indicated on the left of the blots) were immunoprecipated from cells stimulated for various times as shown. These immunoprecipitates were then examined by Western blot analysis for the association of various cellular phosphatases. The results obtained are shown here and the identified phosphatases are indicated on the right of the blots.

We performed pulse chase experiments wherein cells that were pre-equilibrated with ^32^[P]-orthophosphoric acid were chased with excess of non-radioactive phosphate at the time of maximal stimulation with anti-IgG. The target proteins were then immunoprecipitated from cell lysates at various times thereafter, and the extent of phosphorylation determined either by Western blot with specific antibodies, or by autoradiography to monitor the level of radioactive phosphate incorporated. Western blot analysis confirmed that stimulation of cells leads to phosphorylation of all four proteins examined, with the maximally phosphorylated state persisting over the remainder of the experiment (Fig. 3A &3B). This, however, contrasted with the profile obtained for the phosphate-associated radioactivity. A progressive, time-dependent, decline in the specific activity of the radiolabel was detected in all cases (Fig. 3A &3B). Importantly, this dilution in specific activity could be significantly inhibited by the inclusion of phosphatase inhibitors (Fig. 3A &3B). These results, therefore, reveal that the stimulus-induced phosphorylation profile of a signaling intermediate defines a continuum of modulations in the turnover between the phosphorylated and non-phosphorylated states of the target protein.

We also scanned for associations between select signaling intermediates and the protein phosphatases. Five representative signaling intermediates (Akt, ERK-1/2, JNK, PLCγ, and Raf) were immunoprecipitated from lysates of either unstimulated cells, or, cells stimulated either for 10 or 30 min. Immunoprecipitates were then subjected to a Western blot analysis with antibodies directed against the seven phosphatases identified in Figure 1A.

Figure 3D shows that each signaling intermediate associated with multiple cellular phosphatases through a combination of constitutive and dynamic interactions. Thus, Raf was constitutively associated with PP2A and PP1, whereas PLCγ interacted with SHP-1 and SHP-2 (Fig. 3D). In addition, stimulus-dependent modulations were also evident as in the case of PP2A with ERK-1/2, and the recruitment of SHP-1 and HePTP by Raf, and PLCγ respectively (Fig. 3D). This confirms that the phosphorylation status of at least several of the signaling intermediates is regulated by the action of multiple phosphatases both under basal and receptor-activated conditions.

### Altered transcription factor activation and gene expression in response to phosphatase-mediated signal perturbation

**Figure 4:**
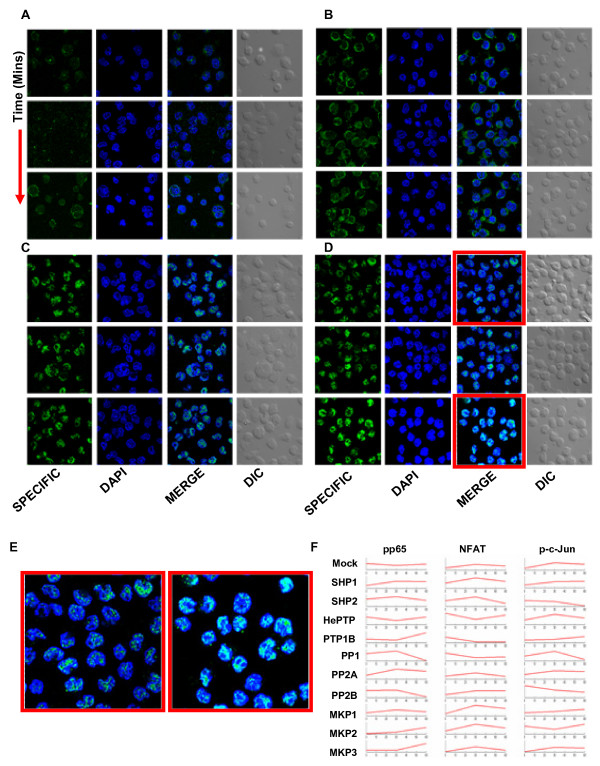
**Differential signal processing results in differential activation of transcription factors**. A20 cells were transfected with phosphatase specific siRNAs or mock siRNA, stimulated for 0, 30 and 60 minutes and were then stained with antibodies specific to transcription factors pp65, NFAT and p-c-jun followed by secondary antibody conjugated to Alaxa488. Cells were also stained with DAPI to locate the nucleus and were then monitored under Nikon TE 2000E microscope equipped with 60×/1.4 NA planapochromat DIC objective lens. Panel A, B and C shows representative fields for pp65, NFAT and p-c-jun respectively. Within each panel, column one shows antibody specific fluorescence, column two shows nuclear staining of the cells by DAPI, third column shows merging of the first two images (to see co-localization) and fourth columns shows white light image of the cells for which fluorescence were measured. The rows in each panel show various time points after stimulation of the cells. Panel D shows similar images for p-c-Jun under SHP1 knockdown condition. Images in column three, panel D bordered red (0 minutes and 60 minutes post stimulation) are enlarged below in Panel E for better depiction of visible differences in co-localization (see text for detail) in stimulated cells. Co-localization coefficients were calculated (for details see Additional Methods) for all the three transcription factors in the nucleus at every time points under all the perturbation conditions. Values measured from a minimum of 15 cells were taken to obtain average co-localization and they are plotted for all the transcription factors under various conditions (Panel F).

To examine the consequences of phosphatase-induced modulations in signaling behavior, we studied activation of a set of three transcription factors (TFs) as a simple and direct readout for modulations in net signal output[11]. These were the p65 subunit of NFκB, NFAT, and the c-Jun subunit of AP-1. TF activation was measured as the extent of nuclear accumulation of the activated form by immunofluorescence-based microscopy [12-17]. Cells treated either with non-silencing, or phosphatase-specific, siRNA were stimulated for 0, 30, or 60 min, and the temporal modulations in the nuclear pool of the three TFs was determined, as a function of phosphatase-depletion (Fig. 4A–D). In the representative example shown, the activation profile of AP1 was significantly altered in cells depleted of SHP-1 (compare Figs. 4C–E).

Figure 4F summarizes the results obtained (Additional files 4 and 5). The observed diversity in the range of activity profiles induced supports that cellular phosphatases play a key role in facilitating the combinatorial processing of signal, thereby leading to multivariate outcomes at the level of TF activation. Consistent with this, depletion either of PP1, PP2A, or SHP1 (representative examples) was also found to influence the BCR-dependent gene expression profile such that a unique pattern was generated in each case (Additional files 6 and 7). Thus, combinatorial modulation of signal processing translates into a multivariate output at the level of transcription factor activation, the outcome of which is then expressed through a diversification in the pattern of gene expression (Additional file 6).

### Defining a signaling axis for transcription factor activation

**Figure 5:**
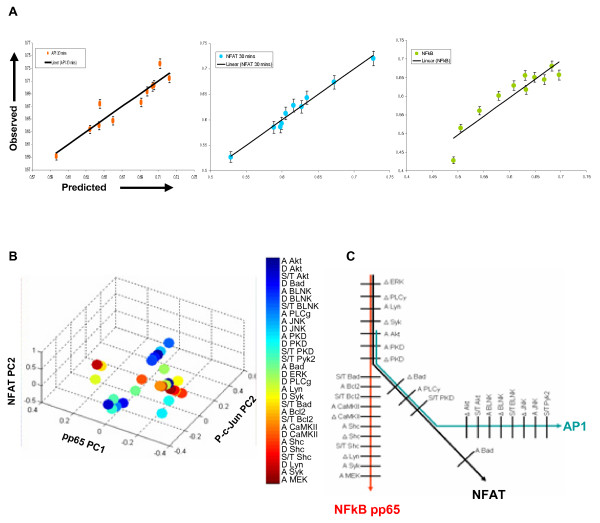
**Signaling axes for transcription factor activation consisting of molecular parameters**. The multivariate data for signaling molecules and transcription factor activation were taken into a partial least square regression model (see text for details). Panel A shows observed versus predicted values for transcription factor activation under various knockdown conditions. The signaling axes for transcription factor activation were determined using our approach to classify signaling parameters on principle component axis according to correlation with TF activation (see text for detail). The overlapping set of signaling parameters are pictorially depicted in panel B in a 3-D plot consisting of PC1 for pp65 and PC2 for NFAT and p-c-Jun model. Response specific signaling parameter tracks are shown in Panel C where unique, common to two and common to all three cellular responses are depicted schematically.

To extract underlying inter-dependencies between the signaling network and TF activation, we used Partial Least Square Regression (PLSR) analysis [18-20]. We trained the PLS model using the signaling parameters as independent variables (X), and the activation profile of the individual TFs as the dependent variables (Y). The signaling variables were determined from the phosphorylation profiles of each intermediate, under each of the siRNA conditions tested. Here, each phosphorylation profile was resolved into three separate parameters, which were the activation rate (S_max_/t_max_; measured as the ratio of the peak activation and the time taken to achieve it), the total area under the phosphorylation curve (A), and the rate of subsequent dephosphorylation (Δ)[10]. Details of the model refinement and validation are provided in Methods (Additional files) and Additional files 8, 9, 10, 11. Figure 5A shows the plot for the observed versus predicted values for all the three TF-activation responses. The prediction accuracy achieved was about 90% for all cases.

We next enlisted all of the principle component axes obtained in the models for each of the TFs. The corresponding signaling parameters were then arranged along these axes in the descending order of their significance to determine whether this produced segregation between those signaling parameters that correlated positively and negatively, with the activation of that particular TF. PC2 yielded this segregation for the models for NFAT and AP1, whereas it was PC1 for the pp65-derived model (Additional file 12). The constituent signaling parameters, and their quantitative distribution in the three TF-specific principle component axis space is shown in Figure 5B. Interestingly, these parameters could be further classified into three groups depending upon whether they were common to all three TFs, common to only any two, or, unique to a given TF (Fig. 5C).

To evaluate the relative sensitivities of the constituent signaling parameters to the individual phosphatases, we examined each signaling axis described in Figure 5C for the extent of phosphatase-induced variation in individual VIPs. These values were expressed as the fold-variation over that obtained in cells treated with non-silencing siRNA, and the results are shown in the form of a pseudo-color diagram in Additional file 13. Individual VIPs that comprise the TF response-axes showed a wide variation in the extent of their sensitivity to the phosphatase-targeted perturbations. As a result, each phosphatase-perturbation yielded its own characteristic fingerprint of VIP values, along each of the three TF activation pathways. That is, each perturbation exerted non-identical effects on the individual signaling parameter tracks for the various TFs, thereby ensuring an output that is multivariate in nature.

Our results highlight two overlapping structural features that complement each other to provide plasticity to the signaling network. At one level, each node was regulated by multiple phosphatases such that both the quantitative and kinetic aspects of its phosphorylation represented the end result of these combined effects. Complementing this was our related finding that each cellular phosphatase, in turn, exerted its influence over multiple nodes of the signaling network. Importantly, this effect was weighted in nature, leading to both quantitative and qualitative variations in the contribution of individual nodes to the net signal output. Thus these combined insights reveal an intricately enmeshed structure for the signaling network, with each node being connected – either directly or indirectly – to several phosphatases on the one hand, and each phosphatase being – in turn – linked to multiple nodes, on the other. This high degree of connectedness allowed for the effects of phosphatase-perturbation to be propagated to a substantial proportion of the nodes of the signaling network. However, given that the 'small world' environment of regulatory phosphatases differed from one node to another, each node experienced this perturbation in distinct ways, leading to a situation where the composition of the output could be diversified in a combinatorial manner. It is this structural feature that sensitizes the signaling network to modulations in component activity, where a given modulation was expressed as a context-unique fingerprint of VIP values. Each such fingerprint in turn translated into variable effects on the TF-specific signaling axes, thus eventually generating a context-unique output in terms of the resulting gene expression profile. The segregation of signaling parameters derived from same node into different response-specific axes extends our earlier findings [10], implicating it as a general mechanism for defining the signal-dependent cellular response. (Please see additional files 14 and 15.)

## Abbreviations

BCR: B Cell Receptor; siRNA: small interfering RNA; anti IgG: Fab2 fragment of Goat anti Mouse IgG; TF: Transcription Factor; PLS: Partial Least Square; PC: Principle component; VIP: Variables in Importance of Projection

## Competing interests statement

The authors declare that they have no competing interests.

## Authors' contributions

The research was conceived and designed by DK and KVSR. DK, RD, ZS and SJ conducted the experiments. RS performed PLS modeling and analysis. DK and KVSR wrote the paper.

## Supplementary Material

Additional File 1 Rationale for the selection of the molecules in this study. The text discuss about how we selected the list of signaling intermediates, phosphatases and transcription factors. Click here for file

Additional File 2 Methods. The text discusses all the methodologies used in this study. Click here for file

Additional File 3 Specific knockdown of phosphatases using specific siRNA. Western blot images are shown depicting specific knockdowns. Click here for file

Additional File 4 Signaling events downstream of BCR following depletion of specific phosphatases. Western blot profiles of signaling intermediate, as obtained under various phosphatase knockdown conditions. Click here for file

Additional File 5 Normalized values for the Western blot data shown in Additional file 4. Quantitated data of western blot profiles. Click here for file

Additional File 6 Microscopy images for transcription factor activation. Confocal microscopy images for the activation of three transcription factors studied here. Click here for file

Additional File 7 Numerical values for the TF activation. Data shows quantitative co-localization coefficient between specific fluorescence and DAPI fluorescence. Click here for file

Additional File 8 Transcription regulation of BCR dependent genes by phosphatases. Pathway specific gene expression data from cells treated with specific siRNAs against individual phosphatases. Click here for file

Additional File 9 Data for GE Superarray experiment. Quantitative values for the gene expression data. Click here for file

Additional File 10 List of VIPs for the three TFs. Variables in importance of projection, for the three TFs activation as listed by the respective PLS model. Click here for file

Additional File 11 Iterative cross-validation of the PLS model. Cross validation of the model for its R2 (variability captured) and Q2 (predictive ability). Click here for file

Additional File 12 Microscopy images of AP1 activation under Signaling intermediate knockdown condition. Confocal microscopy images for the activation of AP1 under new set of perturbations. Click here for file

Additional File 13 Predictive ability of the model for untrained data. Ability of the PLS model to predict AP1 activation under untrained conditions. Click here for file

Additional File 14 Alignment of signaling parameters on PC1 and PC2axes and respective correlation with the three TF activation profile. Functional segregation of signaling parameters on principle component axes along specific TF activation. Click here for file

Additional File 15 Sensitivity of response specific VIPs to perturbations. Sensitivity of response specific VIPs to perturbations. Click here for file
