# Supplementary material for: Cellular phosphatases facilitate combinatorial processing of receptor-activated signals
Source: BMC Res Notes. 2008 Sep 17;1:81. doi: 10.1186/1756-0500-1-81 (PMC2573882; doi:10.1186/1756-0500-1-81)
Supplement: Additional File 1 — Rationale for the selection of the molecules in this study. The text discuss about how we selected the list of signaling intermediates, phosphatases and transcription factors. [file 1756-0500-1-81-S1.doc]

**Rationale for the selection of the molecules in this study**

In order to study the global dynamic behavior and regulatory features there of, we selected representative molecules from various canonical signaling pathways known to be recruited upon BCR stimulation. Thus the signaling molecules included early tyrosine kinases Lyn and Syk along with syk substrate PLCγ2. The PKC family members, activated as a result of PLCγ2 activity (IP3 and Calcium release in addition to production of DAG), were also included into the list. We also included PI3K dependent signaling pathways like Akt pathway and the effector molecules in this pathway Bad and Bcl2. Members of MAP kinase pathway like Raf, MEK1/2, ERK1/2, p38 and JNK were also incorporated. The list was complete with the incorporation of adaptor molecules like BLNK and Shc. Although the molecules selected here belonged to one or other canonical pathway, our intention was to elaborate on the extensive cross talk and resultant regulatory features among them as a function of receptor stimulation.

A total of ten phosphatases were selected for depletion and these included members from the protein tyrosine phosphatase (SHP1, SHP2, HePTP and PTP1B), the Ser/Thr phosphatase (PP1, PP2A and PP2B), and the dual-specificity phosphatase (MKP1, MKP2 and MKP3) families.

Similarly the three transcription factors selected were again to represent various TFs activated by BCR stimulation. Also, the modes of activation of the three TFs are fundamentally different (as discussed in the main text). Our purpose was to obtain insights on global mechanisms involved while processing signal from the receptor to the response level.
