# Supplementary material for: Cellular phosphatases facilitate combinatorial processing of receptor-activated signals
Source: BMC Res Notes. 2008 Sep 17;1:81. doi: 10.1186/1756-0500-1-81 (PMC2573882; doi:10.1186/1756-0500-1-81)
Supplement: Additional File 2 — Methods. The text discusses all the methodologies used in this study. [file 1756-0500-1-81-S2.doc]

**Methods**

**Transfection of cells with siRNA, Stimulation of cells and detection of phosphoproteins**

A20 cells (1 x 10^7^/ml) were stimulated with the F(ab)_2_ fragment of goat anti-mouse IgG at a final concentration of 25 μg/ml in RPMI for a period of up to 60 min. Lysis of cells, immunoprecipitation of signaling intermediates, and the determination of their phopshorylation status by Western blots followed by quantitation as described in Additional files 4 and 5 . Specific siRNAs targeting various phosphatases were procured from Santa Cruz Biotechnmologies with the exception of HePTP, MKP2 and MKP3, which were obtained from Dharmacon Inc. Each siRNA duplex was (10 μg/0.5 x 10^6^ cells) was transfected using the RNAiFect kit (Qiagen), strictly following the protocol recommended by the manufacturer. In parallel experiments we confirmed silencing of the target molecule by a Western blot analysis of the corresponding cell lysates (see Additional file 3). After 48 hours in culture, cells were harvested and then analyzed for the various parameters described in the text.

**Quantitation and Normalization of Western Blot Data.**

Quantitation and normalization of western blots were done as explained earlier (Kumar et al, 2007). Briefly, Western blots obtained were scanned by PDSI personal densitometer from GE biosciences. Scanning was carried out at 50 pixel resolution and 12 bits. These scanned blots were then quantitated by Molecular Dynamics Imagequant software version 5.2. Normalization of data was done as described in Kumar et al 2007.

**Pulse labeling and stimulation of cells:**

A20 cells (10x10^6^) were washed with phosphate free RPMI and then cultured in phosphate free RPMI for 2 hr. ^32^P-orthophosphate was then added to the medium (0.5mCi/ml) and cells were kept at 37^0^C for an additional 5hrs. Subsequent to this, cells were stimulated with anti-IgG for either 3 minutes (for PLCγ and Lyn), or 10 minutes (for ERK1/2 and JNK) after which the medium was immediately replaced with fresh RPMI supplemented with 100-fold excess of cold phosphate, along with anti-IgG. The culture was continued at 37^0^C for the required time points, at the end of which cell pellets were collected and stored in liquid Nitrogen. Subsequent lysis of cells, immunoprecipitation of target proteins, and their analysis by Western blot is as described above.

**Co-Immunoprecipitation and Western blot analysis.**

Lysates were prepared from between 2-5 x 10^7^ cells/group/time point in a 10 mM Tris buffer (pH, 7.5) containing 0.1% NP-40 and 1mM EDTA, along with a cocktail of protease and phosphatase inhibitors. Particulate matter was first removed through centrifugation, following which lysates were pre-cleared with protein A-agarose. Lysates were then incubated with appropriate antibody (0.6 – 1 μg, at 4^o^C for 2h), after which the immune-complexes were precipitated with protein A-sepharose. These immunoprecipitates were resolved on a 10-12% SDS-polyacrylamide gel, transferred onto a nitrocellulose membrane, and then analyzed by Western blot using appropriate antibodies. Detection of the immunoreactive proteins was by enhanced chemiluminescence (Santa Cruz Biotechnology).

**Isolation of RNA and analysis of gene expression with Superarrays.**

Total RNA (1μg) was taken for analysis on a GEArray Q Series Signal Transduction PathwayFinder^TM^ Gene Array, strictly following the manufacturer’s protocol. The autoradiograms obtained were scanned on a Molecular Dynamics Personal Densitometer and the images were analyzed using Imagemaster 2D software.

**Transcription factor binding site prediction:**

Prediction of binding sites for the three selected transcription factors (pp65, NFATc and p-c-Jun) was done using rVISTA programme [21]available freely as a computational tool for prediction of functionally active transcription factors binding sites (TFBS). We used pre-computed whole genome rVISTA mouse database to locate overrepresented TFBS 3000 bp upstream in our gene of interest (at the P-value cutoff 0.006).

## Transcription factor activation and quantitation of data:

A20 cells were transfected with specific siRNAs, and 44-48 hours post transfection cells were harvested and adhered onto BD Cell-Tak ^TM^ coated cover slips. These cells were then stimulated with 25ug/ml of anti-IgG for either 0, 30 or 60 minutes. Subsequent staining and further processing of cells was according to the protocol described earlier[22]. Complete details of the experimental protocol and the methods used for analysis are provided in the following section.

**Measurement of Transcription factor activation using confocal microscopy.**

**Staining Protocol**

Secondary antibodies used in this study were Alexa-Fluor 488 or Phycoerytherin (PE) conjugated anti-Rabbit IgG from (Molecular Probes) and dilutions ranged from 1:100 (for PE conjugated) to 1:1000 (for Alexa-Fluor 488 conjugated). Primary antibodies for detection of transcription factors were phospho p65 (ser 536 ), phospho c-jun (ser 73 ), both from Cell Signaling Technology and NFATc from Santa Cruz Inc. Phospho-specific antibodies were used at 1:250 dilution while NFAT antibody was used at 1:500.

Staining was performed as described (Dimitrova and Gilbert, 1999). To examine co-localization between transcription factors and the nucleus, cells were fixed in 3% paraformaldehyde in PBS for 10min at room temperature followed by quenching with 50mM ammonium chloride for 10min. Fixed cells were permeabilised by incubating with 0.2% Triton X-100 in PBS for 5 minutes. Then cells were incubated in blocking buffer (3% bovine serum albumin and 0.5% Tween20 in PBS) for 20 minutes prior to 45 minutes to 1-hour incubation with respective primary antibodies (diluted in blocking buffer). After incubation with secondary antibody and washing with PBS, the cover slips were incubated with 300nM 4’,6-diamidino-2-phenyl-indole (DAPI) in PBS/T (0.5% Tween 20 in PBS) for 5 minutes followed by three washes with PBS/T. All cover slips were mounted on slides with Antifade (BioRad).

**Image Capturing**

Stained cells were observed with a Nikon TE 2000E microscope equipped with 60X /1.4 NA planapochromat DIC objective lens. DAPI was excited at 405 nm with Blue Diode, Alexa 488 and Phycoerytherin was excited at 488 nm with an argon ion laser. The emissions were recorded through emission filter set 450/35;515/30;605/75. Serial confocal sections (0.5 μm thick) within a z-stack spanning a total thickness of the cell were taken in individual channels Green and Blue or Red and Blue using the motor drive focusing system. The transmission and detector gains were set to achieve best signal to noise ratios and the laser powers were tuned to limit bleaching of fluorescence. The refractive index of the immersion oil used was 1.515 (Nikon). All settings were rigorously maintained for all experiments.

**Image Analysis**

All images were quantified using Image-Pro® Plus version 6.0, a commercially available software package from Media Cybernetics. The merged confocal images were subjected to co-localization analysis to determine the “Overlap Coefficient” proposed by Manders et al, 1993.

Where S1 is signal intensity of pixels in the first channel and S2 is signal intensity of pixels in the second channel.

Atleast 18-20 cells were analyzed in 3 sets of experiments for the co-localization studies. All the images are in the Tiff RGB 24 format and phycoerytherin fluorescence has been given a green pseudocolour. To reduce the unwanted background noise generated by the photomultiplier signal amplification, all the image stacks were treated with two-dimensional filters (Gaussian and sharpening filtering).

**PLS modeling and validation of model.**

PLS modeling and validation of the model was performed following exactly as described earlier [10]. In brief, the PLS model was constructed by including all the signaling variables X and the dependant variables Y (transcription factor activation) using the software suite from UMETRICS, SIMCA-P 11.5. PLS finds the linear (or polynomial) relationship between Y and X (predictor variables) expressed as

*Y = f(X) + E*

PLS is easily understood geometrically, where the matrices X and Y are seen as N points in two different spaces, the X-space with K axes and the Y-space with M axes, K and M being the number of columns in X and Y. PLS modeling consists of simultaneous projections of both the X and Y spaces on low dimensional hyper planes. PLS components are computed by iterative regression to capture the successive signal-response dimensions from the data set. VIP, also known as variable importance in the projection, is the influence of predictor variable x on the response Y and was calculated using the same software. Models obtained were iteratively validated over 20 different iterations. The criteria for validity of the model were R2 and Q2 values for iterated set of models. For details of the validation method, see text and Additional file 11.

**References.**

Dimitrova, D.S., and Gilbert, D.M. (1999). The spatial positioning and replication of chromosomal domains are both established and governed in early G1 phase. Mol. Cell *4*, 983-993.

Manders, M.E., Verbeek, F.J. and Aten, J.A. (1993). Measurement of co-localization of objects in dual-color confocal images, J. Micros. ***169***, pp. 375–382.
