# Supplementary material for: Cellular phosphatases facilitate combinatorial processing of receptor-activated signals
Source: BMC Res Notes. 2008 Sep 17;1:81. doi: 10.1186/1756-0500-1-81 (PMC2573882; doi:10.1186/1756-0500-1-81)
Supplement: Additional File 3 — Specific knockdown of phosphatases using specific siRNA. Western blot images are shown depicting specific knockdowns. [file 1756-0500-1-81-S3.pdf]

Additional File 3

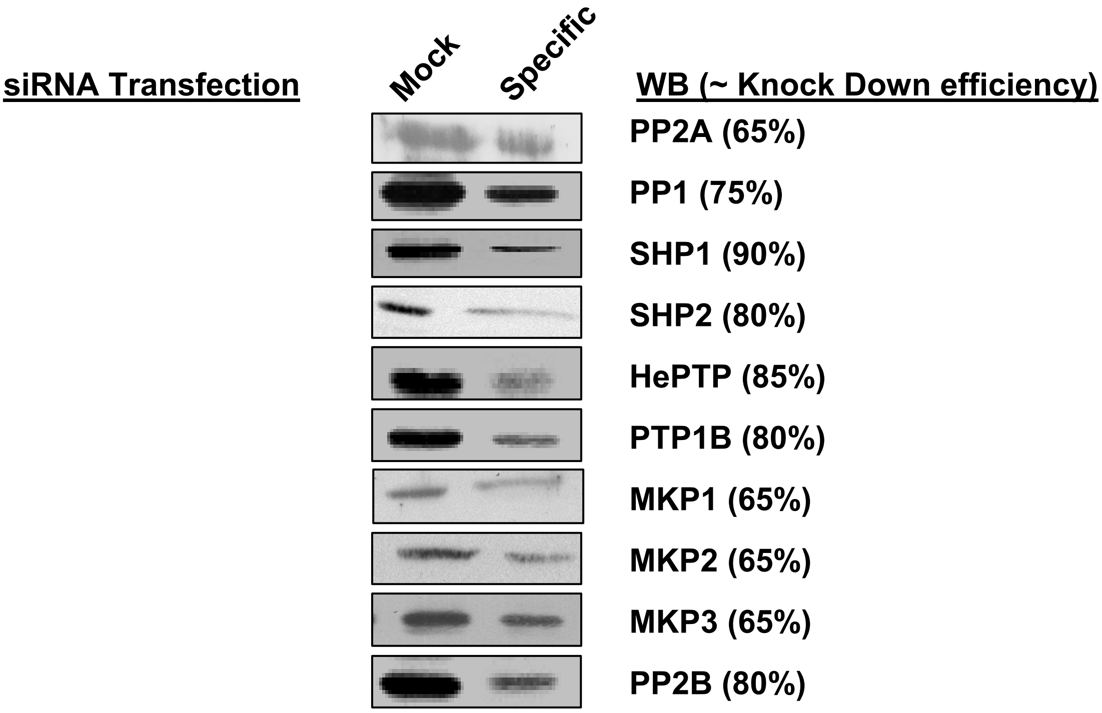

Specific depletion of Phosphatases using siRNAs.

The ten selected phosphatases were individually depleted by using specific siRNA (Santa Cruz and Dharmacon Inc). We used HiPerfect (Qiagen) reagent for transfection of cells with specific siRNAs and we followed the protocol provided by the manufacturer to silence specific genes in suspension cells. For lanes marked mock, a non-specific siRNA (against GFP) was used. The approximate silencing efficiency at the protein level is mentioned for individual molecules.
