# Supplementary material for: Cellular phosphatases facilitate combinatorial processing of receptor-activated signals
Source: BMC Res Notes. 2008 Sep 17;1:81. doi: 10.1186/1756-0500-1-81 (PMC2573882; doi:10.1186/1756-0500-1-81)
Supplement: Additional File 4 — Signaling events downstream of BCR following depletion of specific phosphatases. Western blot profiles of signaling intermediate, as obtained under various phosphatase knockdown conditions. [file 1756-0500-1-81-S4.pdf]

Additional file 4

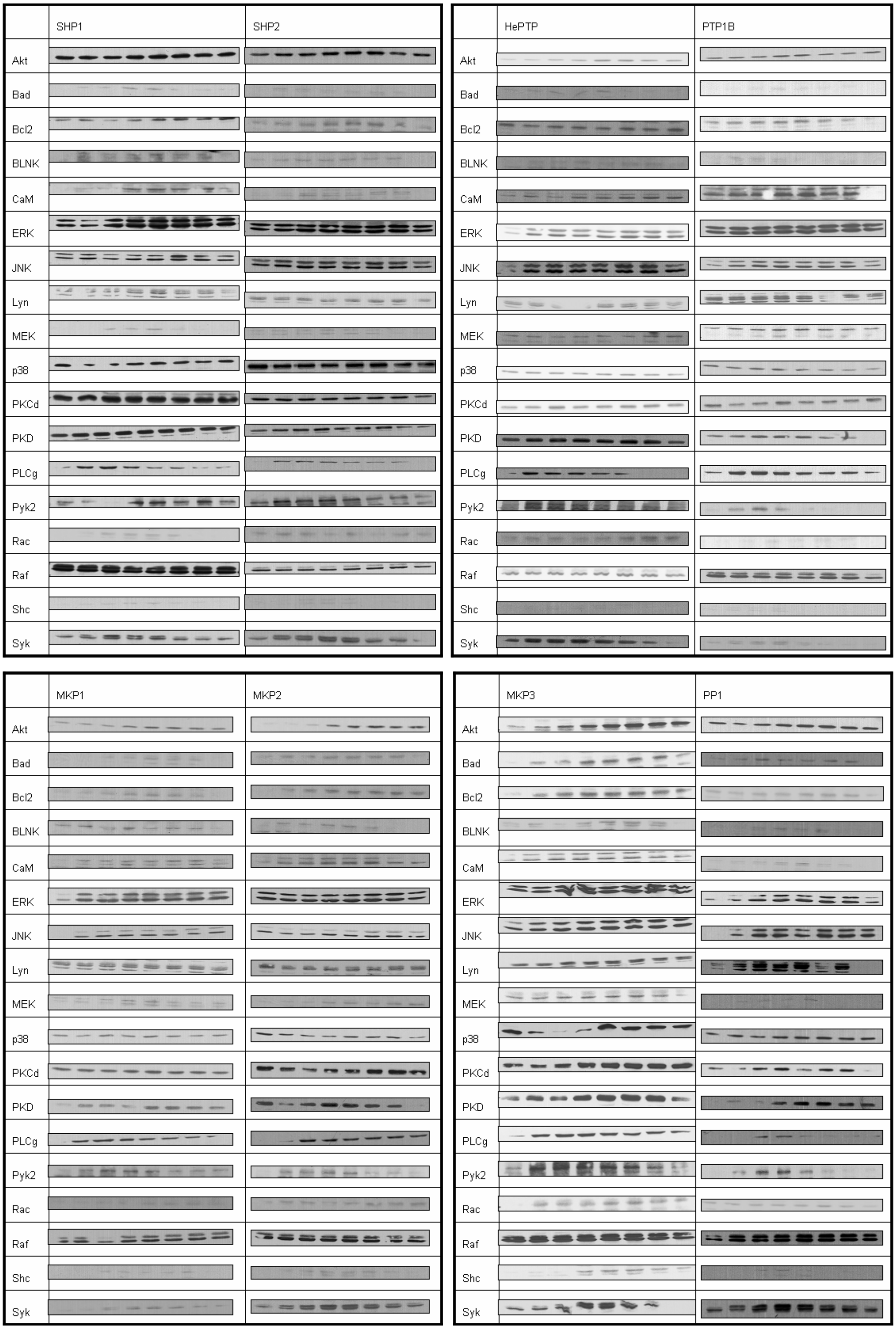

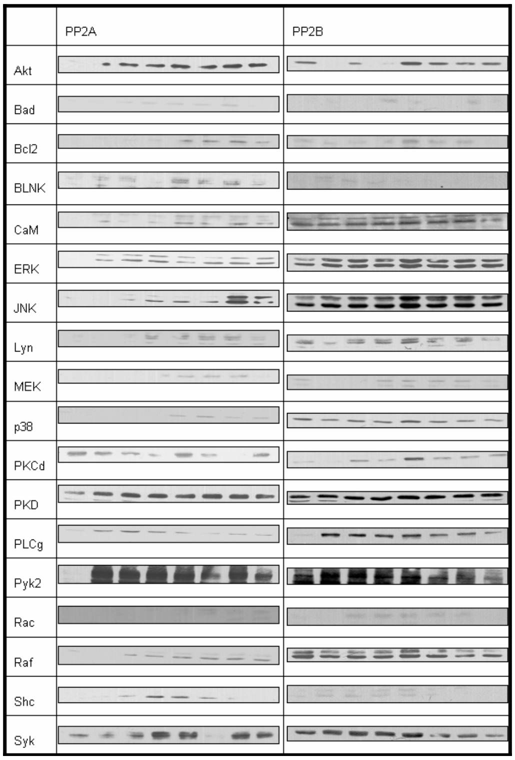

Signaling events downstream of BCR following depletion of specific intermediates

Original scans of Western blots for phosphorylated signaling molecules under all the ten siRNA conditions obtained are shown here. The blots shown here and the normalized data shown in S3 is from one of the three replicates, the standard deviation between the replicates was below 30%. These blots are directly copied from Imagequant. There are a few cases where bands cannot be visualized by the naked eye, due to inhibition in phosphorylation of that molecule. Even in such cases, however, the high sensitivity and resolution of Imagequant allowed for quantitation. In all these latter instances, we confirmed the inhibition of phosphorylation by immunoblotting for the corresponding protein molecule in the same blot, after stripping (following the exact protocol recommended by Cell Signaling Technologies, data not shown).
