# Supplementary material for: Cellular phosphatases facilitate combinatorial processing of receptor-activated signals
Source: BMC Res Notes. 2008 Sep 17;1:81. doi: 10.1186/1756-0500-1-81 (PMC2573882; doi:10.1186/1756-0500-1-81)
Supplement: Additional File 5 — Normalized values for the Western blot data shown in Additional file 4. Quantitated data of western blot profiles. [file 1756-0500-1-81-S5.pdf]

Additional file 5: Normalized values for the Western blot data shown in Additional file 4

Akt	SHP-1	SHP-2	HePTP	PTP1B	PP1	PP2A	PP2B	MKP1	MKP-2	MKP-3
0	200	274	104	123	100	100	202	189	100	138
0.5	278	505	145	136	102	233	210	186	112	267
1	275	507	156	142	125	600	260	185	148	474
3	364	718	220	170	186	637	330	287	321	775
5	419	777	210	187	226	910	434	431	356	1127
10	407	746	191	183	225	960	302	476	534	1334
20	310	465	166	175	210	1006	280	375	500	1096
30	347	443	88	172	172	816	276	325	399	517
Bad										
0	479	100	103	100	100	100	100	100	100	100
0.5	535	300	210	110	188	350	142	130	138	215
1	650	330	280	300	430	722	178	170	184	198
3	885	440	320	690	676	782	324	340	198	426
5	785	550	320	610	584	808	442	720	134	482
10	716	480	260	460	508	856	446	520	126	400
20	631	220	240	320	1176	494	342	410	120	400
30	234	96	120	120	252	245	180	80	70	152
Bcl-2										
0	100	100	100	134	100	100	119	100	100	130
0.5	194	131	103	190	282	124	177	330	100	503
1	214	215	110	212	270	135	185	700	136	995
3	186	268	117	240	294	248	221	840	185	1353
5	127	282	119	246	295	335	325	1180	176	1300
10	123	382	127	167	311	313	217	930	158	1232
20	26	155	135	103	144	160	143	530	148	862
30	14	58	152	71	14	106	86	200	132	444
BLNK										
0	100	220	170	167	100	100	200	120	100	304
0.5	428	420	330	587	610	1640	325	170	754	469
1	366	430	350	870	250	1558	338	163	800	600
3	302	500	520	633	560	3480	1058	167	1692	928
5	244	575	490	656	650	5320	600	127	1823	1020
10	206	576	410	646	1040	4550	404	90	1460	1072
20	124	576	370	640	720	3640	320	67	376	968
30	98	440	125	340	360	1670	183	50	254	228
CamKII										
0	100	100	100	200	240	100	122	180	200	280
0.5	350	116	138	295	304	130	132	570	386	937
1	448	298	202	380	336	180	155	740	472	1018
3	747	264	210	433	384	373	180	740	665	1209
5	566	208	221	459	447	406	214	740	625	1284
10	335	211	328	286	315	618	203	850	756	1400
20	227	167	442	258	292	588	190	800	288	1060
30	167	147	369	188	285	368	112	690	157	663
ERK-1/2										
0	100	182	166	366	100	100	245	112	820	339
0.5	108	192	258	778	174	714	382	620	830	350
1	139	226	272	428	560	1074	465	876	805	353
3	208	298	264	442	662	1121	467	988	808	356
5	222	309	292	462	681	742	560	1076	889	398
10	224	322	266	458	568	634	580	1040	989	446
20	216	330	254	485	522	842	512	993	990	385
30	197	160	138	354	182	850	497	1000	646	368

JNK	SHP-1	SHP-2	HePTP	PTP1B	PP1	PP2A	PP2B	MKP1	MKP-2	MKP-3
0	148	254	375	213	100	100	259	187	131	180
0.5	248	477	380	327	123	100	504	560	124	485
1	203	510	380	398	583	390	565	704	128	667
3	174	453	425	446	650	481	577	654	143	748
5	190	408	575	418	691	538	1030	628	152	822
10	255	487	612	433	713	682	697	752	200	769
20	181	423	622	365	672	3297	670	735	192	717
30	124	193	340	252	525	1516	498	738	154	592
Lyn										
0	645	1409	1458	111	100	100	100	100	138	123
0.5	1074	1346	1385	181	1000	180	130	245	154	235
1	1090	1474	1404	544	6380	202	157	608	173	357
3	2090	1420	1405	424	2539	792	177	614	177	369
5	2726	1404	1528	191	2818	430	221	609	200	401
10	2018	1477	1413	190	2182	411	190	624	205	382
20	982	1425	1474	105	1338	426	160	586	230	304
30	846	935	1307	103	807	280	95	454	222	273
MEK-1/2										
0	100	100	230	100	100	100	100	282	100	429
0.5	100	130	270	166	116	188	116	320	100	464
1	100	154	315	226	153	198	136	303	130	466
3	244	197	220	278	129	241	158	570	194	548
5	346	154	240	255	118	246	216	678	332	582
10	100	86	210	194	107	300	170	388	328	632
20	86	83	220	154	133	221	98	130	310	556
30	82	29	860	112	77	184	58	113	286	176
p38										
0	110	129	106	199	251	211	108	120	132	112
0.5	132	118	113	210	445	240	112	134	133	127
1	123	116	108	227	505	290	127	154	127	128
3	114	103	106	222	594	351	129	148	122	138
5	121	97	113	197	640	527	182	159	120	198
10	92	90	117	156	802	560	118	138	110	128
20	94	69	118	143	643	440	112	116	92	126
30	96	34	104	136	615	332	99	130	80	112
PKC-delta										
0	100	100	145	100	100	100	100	134	100	100
0.5	118	69	158	100	102	77	118	112	102	140
1	172	67	198	80	148	63	192	109	132	142
3	146	53	160	87	202	67	194	149	158	252
5	147	79	157	100	180	93	335	162	180	318
10	123	72	168	85	177	91	230	147	312	309
20	128	30	178	89	179	84	206	144	354	294
30	132	15	162	87	164	79	186	157	222	284
PKD										
0	100	255	116	200	183	154	278	118	163	766
0.5	212	418	234	208	448	913	954	177	342	1485
1	243	505	268	233	563	1755	1252	179	844	2040
3	262	631	308	229	850	1963	1370	185	1412	3396
5	255	607	272	230	2450	2071	1826	188	1134	4284
10	216	516	370	145	2572	2111	1426	176	629	4582
20	196	487	263	124	1314	1938	1260	170	308	4432
30	158	142	168	103	1076	1590	1038	149	220	1176

PLCγ	SHP-1	SHP-2	HePTP	PTP1B	PP1	PP2A	PP2B	MKP-1	MKP-2	MKP-3
0	120	100	100	245	134	300	170	100	100	352
0.5	1248	3470	2417	1212	2170	1384	2682	1900	102	3431
1	1723	4180	1582	1334	3150	1322	2400	2724	1515	4260
3	827	3273	1414	1123	2300	1299	2216	2270	1232	3959
5	295	1859	618	708	1240	1286	2050	1800	1059	3257
10	290	1441	356	518	1110	1170	1238	1400	1002	2892
20	78	1128	242	503	870	962	1118	890	986	2663
30	34	105	125	120	480	890	440	516	732	1873
Pyk2										
0	100	375	415	180	100	100	150	100	150	245
0.5	2068	1272	1330	190	499	1664	345	221	908	1364
1	2415	941	1420	205	995	1578	363	252	1059	2262
3	2810	1000	1672	200	1081	1664	310	235	1204	1905
5	3895	782	888	178	742	1596	292	217	816	1776
10	3158	313	868	142	363	1466	190	203	325	1368
20	2790	350	844	130	322	1302	159	225	392	776
30	1183	126	294	123	205	962	75	208	385	544
Rac										
0	100	100	190	100	100	242	100	100	100	132
0.5	145	138	186	140	144	332	286	300	360	212
1	425	140	188	322	300	402	828	350	365	193
3	374	139	190	865	428	367	828	450	422	185
5	318	141	190	530	540	367	792	500	468	171
10	276	126	292	520	644	345	742	465	534	167
20	210	124	497	508	1336	213	586	430	486	166
30	108	102	326	402	436	101	128	386	480	138
Raf										
0	343	100	116	122	100	100	170	103	152	118
0.5	312	138	114	111	309	103	133	121	182	122
1	323	140	121	109	300	106	130	128	182	123
3	284	139	123	106	354	170	130	144	186	129
5	260	141	124	103	430	188	126	135	200	129
10	270	126	150	118	410	183	82	150	185	137
20	232	124	148	115	368	182	74	179	126	133
30	202	102	133	112	245	178	63	182	117	98
Shc										
0	200	156	100	100	100	100	100	100	100	100
0.5	270	160	160	102	458	133	1136	440	305	316
1	364	176	557	118	1018	203	1140	800	385	344
3	322	312	657	488	1120	832	1130	1000	1085	2058
5	319	348	470	266	757	612	1120	920	1400	2714
10	304	240	318	214	254	380	486	900	1270	2728
20	150	160	102	142	140	147	450	906	1050	1900
30	138	60	54	55	109	108	390	420	745	916
Syk										
0	100	100	100	446	100	100	332	123	162	388
0.5	160	251	314	753	146	162	408	207	347	471
1	328	290	334	778	348	197	466	347	692	744
3	202	461	340	796	643	234	495	344	1338	1476
5	233	414	206	390	436	265	631	321	1091	1552
10	119	185	110	229	296	250	236	312	753	1150
20	101	139	33	93	266	171	186	200	534	515
30	80	32	18	40	112	142	106	148	285	224
