# Supplementary material for: Cellular phosphatases facilitate combinatorial processing of receptor-activated signals
Source: BMC Res Notes. 2008 Sep 17;1:81. doi: 10.1186/1756-0500-1-81 (PMC2573882; doi:10.1186/1756-0500-1-81)
Supplement: Additional File 6 — Microscopy images for transcription factor activation. Confocal microscopy images for the activation of three transcription factors studied here. [file 1756-0500-1-81-S6.pdf]

Additional file 6: Microscopy images for transcription factor activation

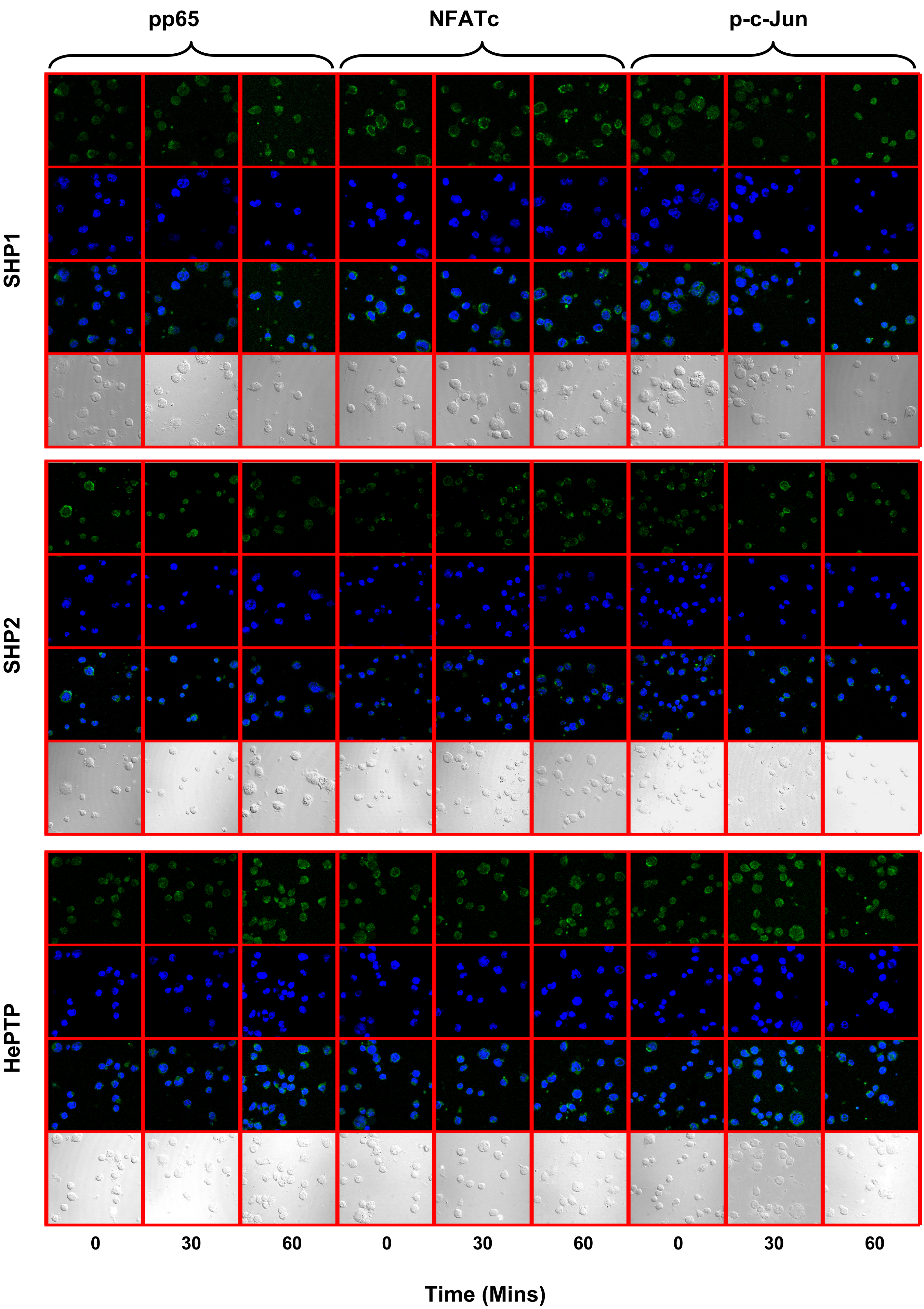

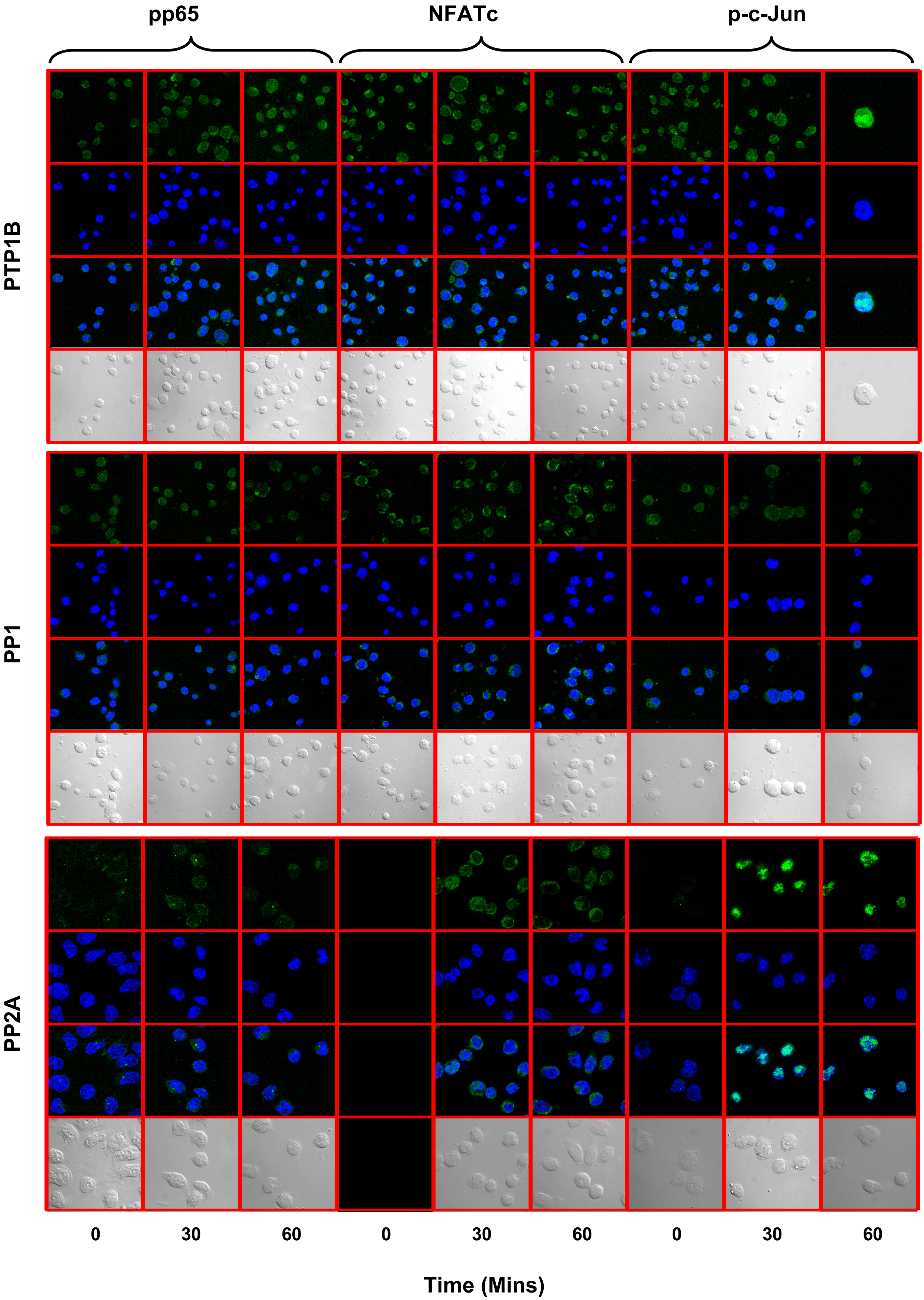

pp65
NFATc
p-c-Jun
PTP1B
PP1
PP2A
0
30
60
0
30
60
0
30
60
Time (Mins)

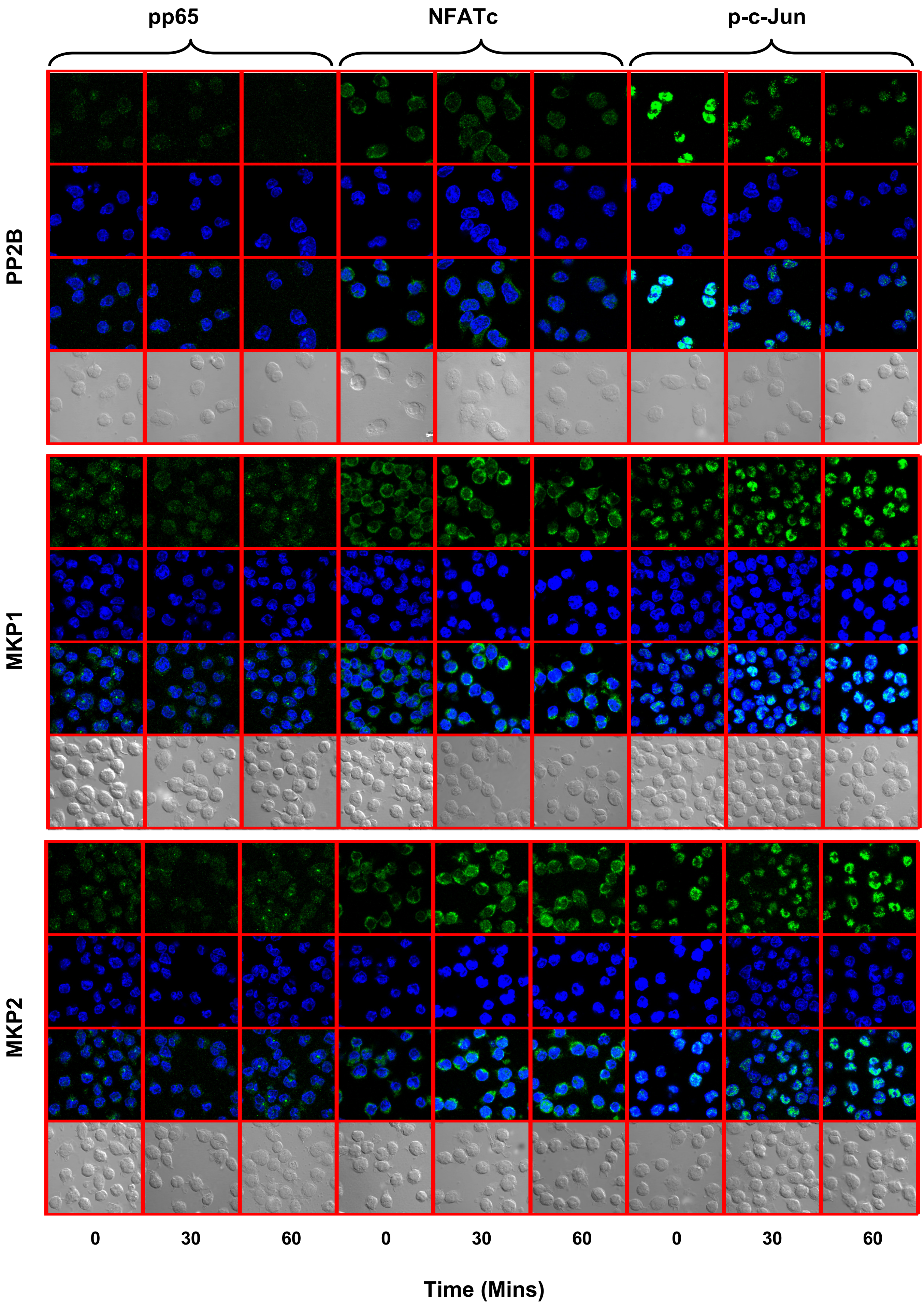
pp65
NFATc
p-c-Jun
PP2B
MKP1
MKP2
0
30
60
0
30
60
0
30
60
Time (Mins)

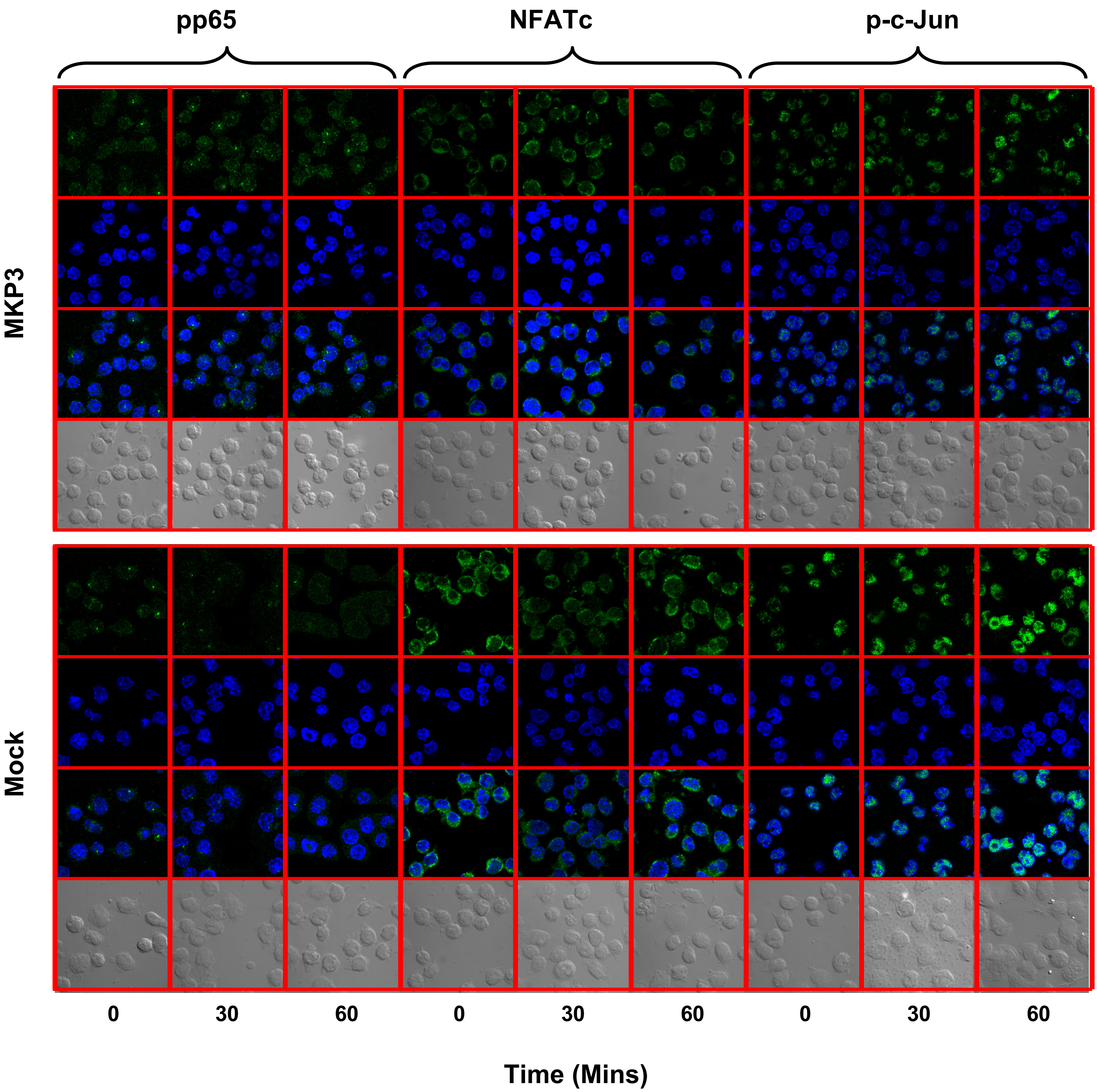

Additional file 6: Microscopy images for Transcription factor activation
Figure shows antibody specific fluorescence (row 1, green), nuclear staining of the cells by DAPI (row 2, blue), merging of the first two images (to see co-localization, row 3) and fourth row shows DIC images of the cells for which fluorescence were measured. The rows in each panel show various time points (0, 30 and 60 minutes) after stimulation of the cells. The experimental details are provided in the Additional files 2.
