# Supplementary material for: Cellular phosphatases facilitate combinatorial processing of receptor-activated signals
Source: BMC Res Notes. 2008 Sep 17;1:81. doi: 10.1186/1756-0500-1-81 (PMC2573882; doi:10.1186/1756-0500-1-81)
Supplement: Additional File 7 — Numerical values for the TF activation. Data shows quantitative co-localization coefficient between specific fluorescence and DAPI fluorescence. [file 1756-0500-1-81-S7.pdf]

Additional flie 7: Numerical Values for the TF activation data

	pp65			NFAT			p-c-Jun			
Time (Mins)	0	30	60	0	30	60	0	30	60	
Mock	0.51	0.48	0.50	0.47	0.59	0.53	0.61	0.66	0.65	Mean
	0.05	0.04	0.02	0.04	0.04	0.05	0.04	0.03	0.05	SD
SHP1	0.54	0.62	0.61	0.54	0.59	0.56	0.68	0.70	0.71	Mean
	0.06	0.04	0.05	0.05	0.04	0.05	0.05	0.10	0.04	SD
SHP2	0.64	0.73	0.59	0.60	0.63	0.57	0.65	0.64	0.60	Mean
	0.05	0.04	0.05	0.04	0.06	0.04	0.06	0.05	0.04	SD
HePTP	0.61	0.58	0.61	0.65	0.59	0.63	0.67	0.69	0.68	Mean
	0.05	0.04	0.05	0.05	0.06	0.04	0.05	0.05	0.06	SD
PTP1B	0.63	0.62	0.69	0.66	0.64	0.64	0.69	0.71	0.81	Mean
	0.05	0.04	0.05	0.05	0.05	0.05	0.03	0.05	0.00	SD
PP1	0.66	0.69	0.60	0.57	0.53	0.53	0.59	0.63	0.56	Mean
	0.03	0.05	0.04	0.05	0.06	0.09	0.08	0.03	0.06	SD
PP2A	0.36	0.47	0.43	0.50	0.61	0.49	0.38	0.70	0.65	Mean
	0.03	0.05	0.04	0.06	0.08	0.09	0.03	0.03	0.06	SD
PP2B	0.41	0.42	0.31	0.50	0.63	0.63	0.79	0.65	0.58	Mean
	0.04	0.04	0.02	0.09	0.06	0.06	0.04	0.05	0.05	SD
MKP1	0.51	0.52	0.51	0.54	0.67	0.62	0.69	0.74	0.81	Mean
	0.03	0.04	0.04	0.03	0.06	0.07	0.05	0.03	0.03	SD
MKP2	0.50	0.51	0.55	0.50	0.72	0.60	0.73	0.68	0.76	Mean
	0.03	0.04	0.04	0.04	0.06	0.06	0.04	0.06	0.07	SD
MKP3	0.51	0.51	0.54	0.42	0.59	0.46	0.55	0.59	0.58	Mean
	0.04	0.05	0.04	0.06	0.06	0.07	0.06	0.04	0.03	SD
