# Supplementary material for: Cellular phosphatases facilitate combinatorial processing of receptor-activated signals
Source: BMC Res Notes. 2008 Sep 17;1:81. doi: 10.1186/1756-0500-1-81 (PMC2573882; doi:10.1186/1756-0500-1-81)
Supplement: Additional File 8 — Transcription regulation of BCR dependent genes by phosphatases. Pathway specific gene expression data from cells treated with specific siRNAs against individual phosphatases. [file 1756-0500-1-81-S8.pdf]

Additional file 8: Transcriptional regulation of BCR dependent genes by Phosphatases

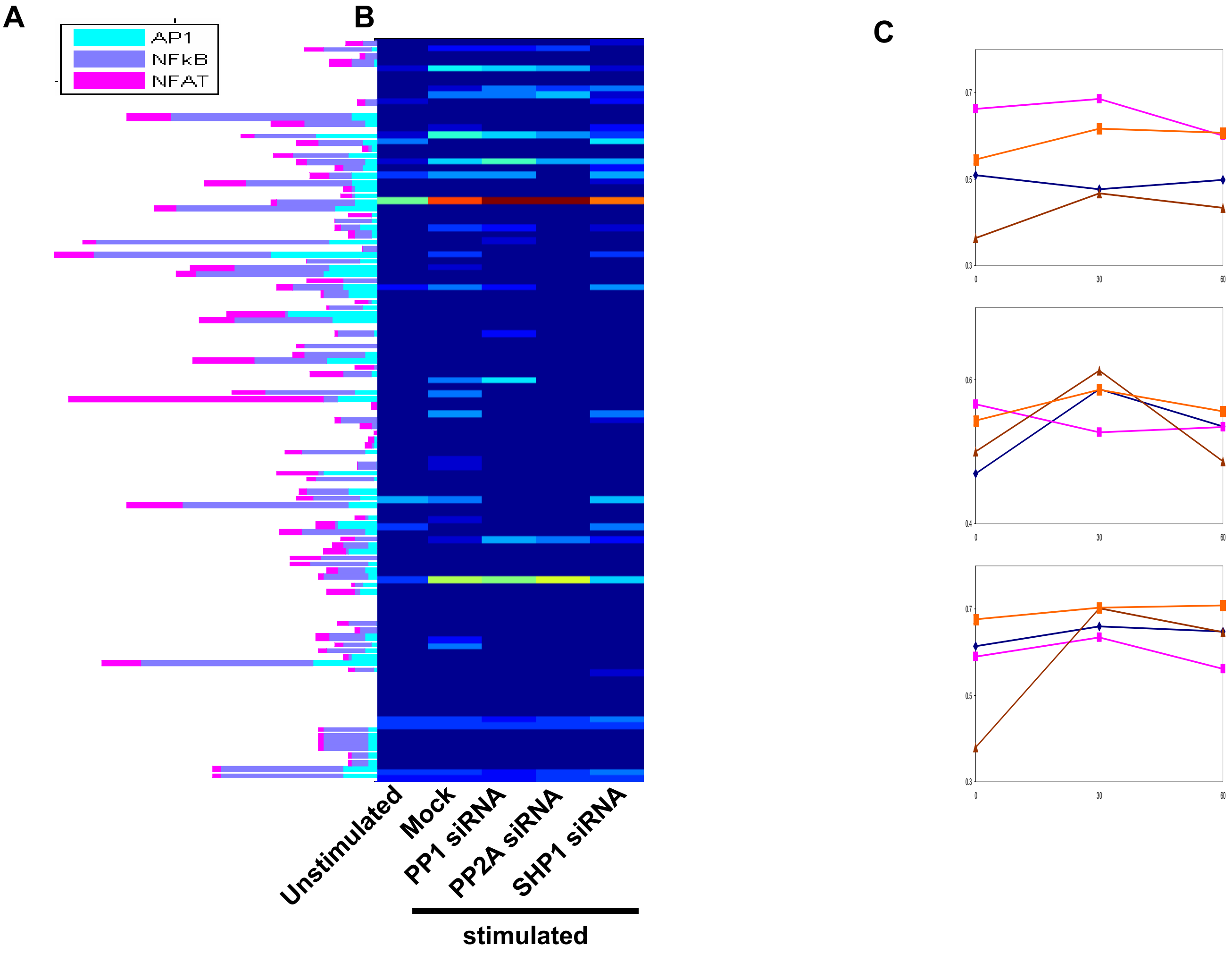

Additional file 8: Transcriptional regulation of BCR dependent genes by Phosphatases. Panel A shows the binding sites for the three transcription factors studied in Figure 4 in a set of 100 genes (see text for detail) as determined by rVista programme. Panel B shows expression profile obtained in a microarray experiments consisting of above mentioned genes on RNA isolated from either unstimulated cells, or on anti-IgG stimulated cells that had first been treated either with non-silencing siRNA (Mock) or with siRNA specific for the indicated phosphatases. The color bar on the right corresponds to the intensity of gene expression. Panel C shows relative activation profiles of the three transcription factors (pp65, NFAT and AP1 from top to bottom) under mock (Pink lines) or phosphatase specific siRNA treatments (PP1, PP2A and SHP1 as x, y and z lines respectively).
