# Supplementary material for: Cellular phosphatases facilitate combinatorial processing of receptor-activated signals
Source: BMC Res Notes. 2008 Sep 17;1:81. doi: 10.1186/1756-0500-1-81 (PMC2573882; doi:10.1186/1756-0500-1-81)
Supplement: Additional File 9 — Data for GE Superarray experiment. Quantitative values for the gene expression data. [file 1756-0500-1-81-S9.pdf]

Additional file 9: Data for GE Superarray experiment

	ohr	1hr	1hr-PP1	1hr-PP2A	1hr-SHP1
Pzp	1.85	1.66	0	0	2.458
Atf2	0.87	3.34	3.676	4.642	1.446
Bax	0	0	0	0	0
Bcl2	0	0	0	0	0
Bcl2a1d	2.35	11.88	9.978	7.688	2.83
Bcl2l	0	0	0	0	0
Birc1a	0	0	0	0	0
Birc1b	0.27	2.63	5.092	4.74	5.026
Birc2	1.76	5.63	5.6	8.582	2.842
Birc3	2.57	0.99	0	0	3.106
Bmp2	0	0	0	0	0
Bmp4	0	0	0	0	0
Brca1	0	0	0	0	0
Ccnd1	1.47	2.22	0	0	3.958
CD5	2.07	13.29	9.468	6.336	4.494
Cdk2	5.04	1.96	0	0	10.908
Cdkn1a	0	0	0	0	0
Cdkn1b	0	0	0	0	0
Cdkn1c	2.98	9.01	14.134	7.454	7.964
Cdkn2a	0.69	1.64	0	0	3.012
Cdkn2b	4.33	6.77	6.964	0.342	7.496
Cdkn2c	0.53	0	0	0	2.856
Cdkn2d	0	0	0	0	0
Cdx1	0	0	0	0	0
Cebpb	16.17	46.23	78.996	127.91	41.35
Csf2	1.67	0	0	0	0
Csnb	0	0	0	0	0
Ctsd	0	0	0	0	0
Cyp19a1	1.55	4.12	3.36	0	2.442
Egfr	0	0	0	0	0
Egr1	0	1.33	2.174	0	0
Ei24	0	0	0	0	0
En1	1.63	4.31	1.974	0	4.254
Fasn	0	0	0	0	0
Fn1	0.11	2.79	0.31	0	1.6
Fos	0	0	0	0	0.298
Foxa2	0	0	0	0	0
Gadd45a	3.03	5.14	3.488	0	6.284
Gys1	0.51	0.74	0	0	1.978
Hhip	0	0	0	0	0
Hk2	0.21	0.9	0	0	0.808
Hoxa1	0.38	1.26	0	0	0
Hoxb1	0	0	0	0	0
Hsf1	0	0	0	0	0
Hspb1	0	0	3.072	0	0
HSP90-Rik	0	0	0	0	0
Icam1	0	0	0.968	0	0
Igfbp3	0	0	0	0	0
Il2	0	0	0	0	0
Il2ra	0.05	0	0	0	0
Il4	0	0	0	0	0
Il4ra	0	5.75	10.45	0.162	0
Irf1	0	0	0	0	0

Jun	0.05	5.59	1.59	0	0.548
Junb	0.1	0	0	0	0.856
Klkb1	0	0.62	0	0	0
Klk6	1.99	6.31	1.9	0	5.718
Lep	0.08	1.76	0.594	0	2.124
Lta	0.08	0	0	0	0
Mdm2	0	0	0	0	0
Mmp10	0	0	0	0	0.822
mmp7	0	0	0	0	0
Myc	0	0	0	0	0
Tnepai	0.95	2.79	1.432	0	0.884
Nfkb1	0	2.05	0	0	0
Nfkbia	0	0	0	0	0
Nos2	0	0	0	0	0
Odc	0	0	0	0	0.66
Pgr	0	0	0	0	0
Prkca	7.26	5.42	0.964	0	8.53
Prkce	0	0	0	0	1.522
Ptch	0	1.76	0	0	0.378
Ptch2	1.63	2.33	0	0	0
Pten	4.23	0	0	0	5.784
Ptgs2	0	0	0	0	0
Rbp1	0.94	2.5	7.108	5.782	3.016
Rbp2	0	0	1.178	0	0
Ccl2	0	0	0	0	0
Cxcl9	0	0	0.356	0	1.412
Sele	0	0	0	0	0
Selp	1.11	1.67	0	0	0.626
Stra6	4.84	19.38	17.25	23.044	9.59
Stra8	0	0	0	0	0
Tnf	0	0	0.464	0	0
Tnfrsf10b	0	0	0.134	0	0
Tnfrsf6	0	0	0	0	0
Tnfsf6	0	0	0	0	0
Trfr	1.33	0	0	0	1.748
Trim25	0	0	0	0	1.064
Trp53	0	0	0	0	0
Vcam1	0.54	3.41	0	0	1.746
Wisp1	0.17	5.54	0.75	0	0.788
Wisp2	0	0	0	0	0
Wnt1	0	0	0	0	0
Wnt2	0	0.76	0.212	0	0
Wsb1	0	0	0	0	2.678
PUC18	0	0	0	0	0
PUC18	0	0	0	0	0
PUC18	0	0	0	0	0
Blank	0	0	0	0	0
Blank	0	0	0	0	0
Blank	0	0	0	0	0
Gapd	4.55	4.67	3.99	4.5	5.45
Gapd	4.16	4.04	4.382	4.26	4.052
Ppia	1.47	1.47	1.47	1.47	1.47
Ppia	1.4	1.4	1.4	1.4	1.4
Ppia	1.19	1.19	1.19	1.19	1.19
Ppia	1.58	1.58	1.58	1.58	1.58
Rpl13a	0.38	0.38	0.38	0.38	0.38
Rpl13a	0.48	0.48	0.48	0.48	0.48
Actb	4.1	4.72	3.8	4.12	5.178
Actb	3.39	3.75	3.67	4.3	4.554
