# Supplementary material for: Cellular phosphatases facilitate combinatorial processing of receptor-activated signals
Source: BMC Res Notes. 2008 Sep 17;1:81. doi: 10.1186/1756-0500-1-81 (PMC2573882; doi:10.1186/1756-0500-1-81)
Supplement: Additional File 10 — List of VIPs for the three TFs. Variables in importance of projection, for the three TFs activation as listed by the respective PLS model. [file 1756-0500-1-81-S10.pdf]

Additional file 10: List of VIPs for the three Transcription Factors

pp65		NFAT		AP1	
Var ID (Primary)	VIP Value	Var ID (Primary)	VIP Value	Var ID (Primary)	VIP Value
plcg decay rate	1.7823	erk decay rate	1.63503	pkd area	1.71789
plcgarea	1.76275	pkd area	1.57247	pkd decay rate	1.63817
shc area	1.69295	pkd decay rate	1.54918	jnk decay rate	1.42545
syk decay rate	1.62744	akt area	1.49976	plcgarea	1.42488
shc decay rate	1.62581	bad area	1.45844	Blnkdecay rate	1.42304
pkd area	1.58098	baddecay rate	1.44974	plcg Smax/Tmax	1.4108
lyn area	1.52334	lyn area	1.43893	BLNK area	1.39447
lyn decay rate	1.50458	plcg decay rate	1.42881	akt decay rate	1.3896
bcl2 area	1.45844	syk decay rate	1.42363	jnk area	1.3607
syk area	1.42722	erk area	1.28594	aktSmax/Tmax	1.36017
pkd Smax/Tmax	1.42567	lyn Smax/Tmax	1.23271	blnkSmax/Tmax	1.3576
pkd decay rate	1.37384	p38 decay rate	1.23006	akt area	1.34789
pkcdarea	1.34666	raf area	1.21288	pyk2 Smax/Tmax	1.2992
akt area	1.27218	p38 area	1.20444	plcg decay rate	1.23392
bad Smax/Tmax	1.27188	shc decay rate	1.1898	raf decay rate	1.23336
Camkii area	1.27073	akt decay rate	1.18639	baddecay rate	1.19163
shc Smax/Tmax	1.25824	plcgarea	1.1539	pkd Smax/Tmax	1.17685
erk area	1.22989	shc area	1.14792	p38 area	1.11326
erk decay rate	1.12824	syk area	1.10011	bad Smax/Tmax	1.11064
bcl2 Smax/Tmax	1.05939	Camkii area	1.07707	erk area	1.06494
jnk area	1.05741	pkcd decay rate	1.07676	p38 decay rate	1.04027
mek area	1.02681	bcl2 Smax/Tmax	1.03227	erk Smax/Tmax	1.02373
pkcd decay rate	0.923442	RAC Smax/Tmax	1.02736	bad area	0.968795
mek decay rate	0.894869	pyk2 area	1.01453	syk area	0.943186
bcl2 decay rate	0.888947	BLNK area	1.00668	pkcdarea	0.916098
plcg Smax/Tmax	0.861922	jnk area	1.00413	jnk Smax/Tmax	0.908837
akt decay rate	0.804701	pyk2 decay rate	0.995797	shc area	0.900897
raf area	0.774882	pkd Smax/Tmax	0.940487	pyk2 decay rate	0.8649
raf Smax/Tmax	0.713584	Blnkdecay rate	0.932326	shc decay rate	0.863323
pyk2 area	0.711794	bcl2 area	0.912873	Camkii area	0.860539
RAC decay rate	0.709796	jnk decay rate	0.872587	erk decay rate	0.850372
camkii Smax/Tmax	0.672227	lyn decay rate	0.823212	shc Smax/Tmax	0.850057
RAC Smax/Tmax	0.618565	mek decay rate	0.82032	lyn Smax/Tmax	0.841769
jnk decay rate	0.606757	bcl2 decay rate	0.816838	bcl2 area	0.779015
BLNK area	0.586922	mek area	0.796387	pkcd Smax/Tmax	0.754242
p38 Smax/Tmax	0.572269	RAC decay rate	0.78661	raf Smax/Tmax	0.720455
erk Smax/Tmax	0.565799	pyk2 Smax/Tmax	0.754048	lyn area	0.70367
pkcd Smax/Tmax	0.560479	raf decay rate	0.751584	mek area	0.693626
raf decay rate	0.496616	erk Smax/Tmax	0.737285	syk Smax/Tmax	0.66867
camkiidecay rate	0.480196	pkcdarea	0.73563	pkcd decay rate	0.664176
lyn Smax/Tmax	0.445035	camkii Smax/Tmax	0.694055	RAC Smax/Tmax	0.656719
pyk2 decay rate	0.441619	aktSmax/Tmax	0.550519	camkiidecay rate	0.654571
blnkSmax/Tmax	0.437742	RACarea	0.525354	p38 Smax/Tmax	0.650796
bad area	0.378897	plcg Smax/Tmax	0.507348	syk decay rate	0.644145
Blnkdecay rate	0.343287	syk Smax/Tmax	0.445309	bcl2 Smax/Tmax	0.62916
aktSmax/Tmax	0.276383	bad Smax/Tmax	0.397933	pyk2 area	0.568309
syk Smax/Tmax	0.22959	mek Smax/Tmax	0.367627	raf area	0.567327
pyk2 Smax/Tmax	0.20052	raf Smax/Tmax	0.34426	RACarea	0.548988
RACarea	0.195903	p38 Smax/Tmax	0.272776	mek Smax/Tmax	0.378855
mek Smax/Tmax	0.178846	camkiidecay rate	0.271791	lyn decay rate	0.332804
jnk Smax/Tmax	0.178621	shc Smax/Tmax	0.242533	mek decay rate	0.284921
p38 area	0.06206	blnkSmax/Tmax	0.242497	bcl2 decay rate	0.281702
baddecay rate	0.024072	pkcd Smax/Tmax	0.158455	RAC decay rate	0.25814
p38 decay rate	0.000287	jnk Smax/Tmax	0.081015	camkii Smax/Tmax	0.051383
