# Supplementary material for: Cellular phosphatases facilitate combinatorial processing of receptor-activated signals
Source: BMC Res Notes. 2008 Sep 17;1:81. doi: 10.1186/1756-0500-1-81 (PMC2573882; doi:10.1186/1756-0500-1-81)
Supplement: Additional File 11 — Iterative cross-validation of the PLS model. Cross validation of the model for its R2 (variability captured) and Q2 (predictive ability). [file 1756-0500-1-81-S11.pdf]

Additional file 11: Iterative Cross-validation of the PLS model

pp65

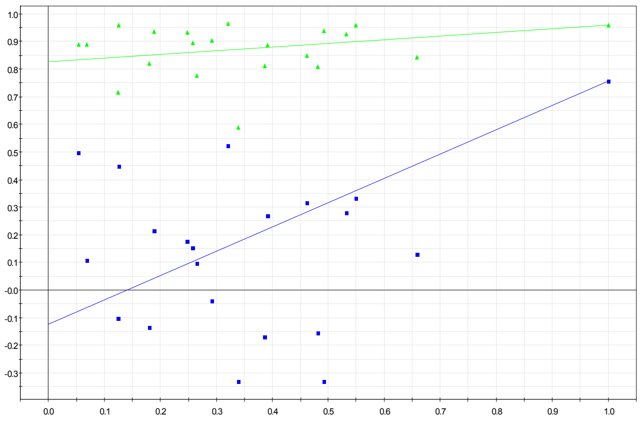

NFAT

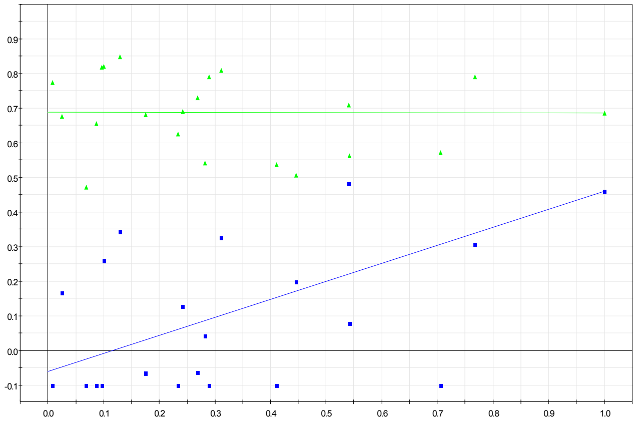

AP1

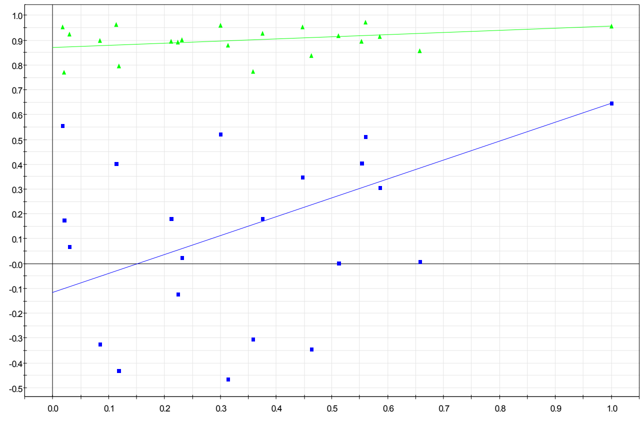

Additional file 11: Iterative Cross-validation of the PLS model

Each plot represents the cross validation by iteratively retraining the model parameters. We performed 20 different iteration for the given data set. The R2Q2 regression plot for the iterated models, as shown in the figure S7, shows none of the iterated model set has a value higher than the base model for R2 or Q2 and the Q2 plot intercepts the Y axis well below zero. This confirms validity of the base model.
