# Supplementary material for: Cellular phosphatases facilitate combinatorial processing of receptor-activated signals
Source: BMC Res Notes. 2008 Sep 17;1:81. doi: 10.1186/1756-0500-1-81 (PMC2573882; doi:10.1186/1756-0500-1-81)
Supplement: Additional File 12 — Microscopy images of AP1 activation under Signaling intermediate knockdown condition. Confocal microscopy images for the activation of AP1 under new set of perturbations. [file 1756-0500-1-81-S12.pdf]

Additional file 12: Microscopy images of AP1 activation under Signaling intermediate knockdown condition

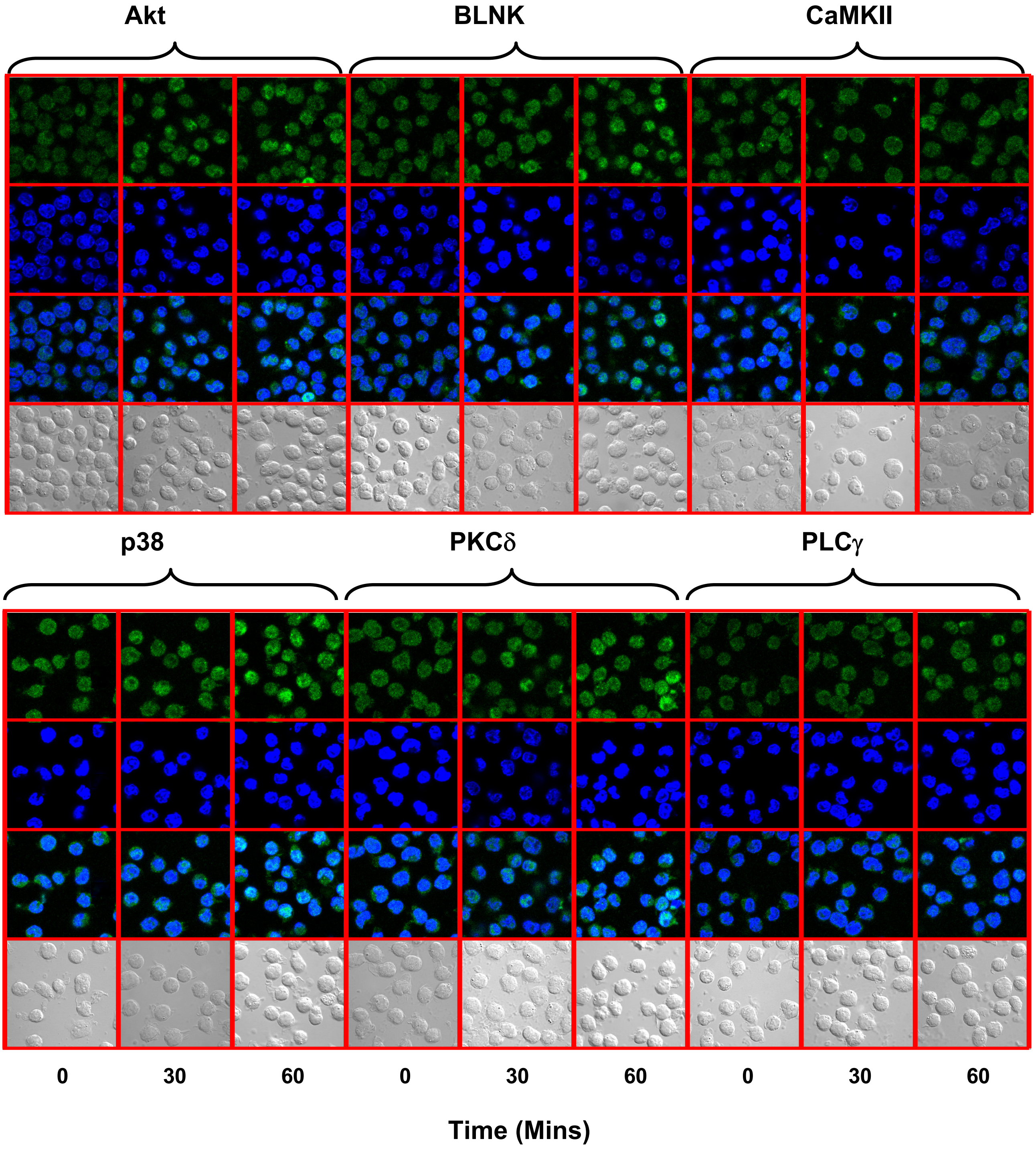

Additional file 12: Microscopy images of AP1 activation under signaling intermediate knockdown condition

Figure shows antibody specific fluorescence (row 1, green), nuclear staining of the cells by DAPI (row 2, blue), merging of the first two images (to see co-localization, row 3) and fourth row shows DIC image of the cells for which fluorescence were measured. The rows in each panel show various time points (0, 30 and 60 minutes) after stimulation of the cells. The experimental details are provided in the Supplementary text, Materials and Methods section.
