# Supplementary material for: Cellular phosphatases facilitate combinatorial processing of receptor-activated signals
Source: BMC Res Notes. 2008 Sep 17;1:81. doi: 10.1186/1756-0500-1-81 (PMC2573882; doi:10.1186/1756-0500-1-81)
Supplement: Additional File 13 — Predictive ability of the model for untrained data. Ability of the PLS model to predict AP1 activation under untrained conditions. [file 1756-0500-1-81-S13.pdf]

Additional file 13: Predictive ability of the model for untrained data

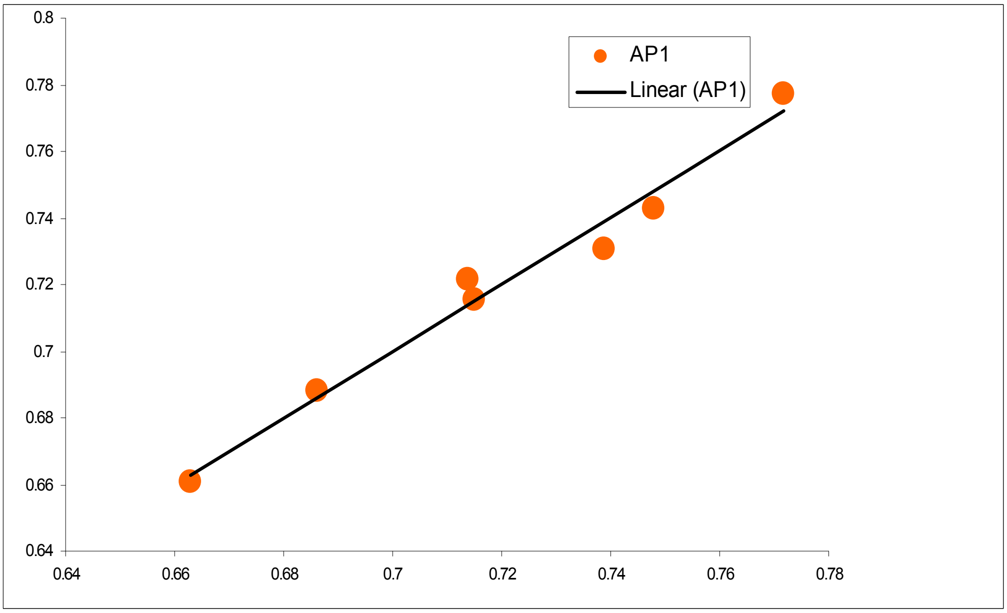

Additional file 13: Predictive ability of the model for untrained data
Untrained data set for signaling intermediates was taken from our earlier work (Kumar et al, 2007). We generated data for AP1 activation (c-jun phosphorylation profile) in a similar fashion as was done for the phosphatase knockdowns (See Materials and Methods section and Supplementary text for the experimental details). The data set was taken into the PLS model, Figure shows a perfect regression line between the observed and predicted values for transcription factor activation under signaling intermediate knockdown conditions.
