# Supplementary material for: Cellular phosphatases facilitate combinatorial processing of receptor-activated signals
Source: BMC Res Notes. 2008 Sep 17;1:81. doi: 10.1186/1756-0500-1-81 (PMC2573882; doi:10.1186/1756-0500-1-81)
Supplement: Additional File 14 — Alignment of signaling parameters on PC1 and PC2axes and respective correlation with the three TF activation profile. Functional segregation of signaling parameters on principle component axes along specific TF activation. [file 1756-0500-1-81-S14.pdf]

Additional file 14: Alignment of signaling parameters on PC1 and PC2 axes and respective correlation with the three TF activation profile

AP1

Correlation	Var ID (Primary)	PC1
0.619220029	blnkSmax/Tmax	0.0129658
0.227311198	jnk decay rate	-0.257772
0.08452165	akt decay rate	-0.312144
0.039849629	BLNK area	-0.299696
0.035653873	jnk area	-0.304618
0.004325512	Blnkdecay rate	-0.288818
-0.295971068	baddecay rate	-0.131637
-0.384493909	pkd decay rate	-0.359694
-0.405308952	akt area	-0.37792
-0.624633789	aktSmax/Tmax	-0.164964
-0.645311466	pyk2 Smax/Tmax	-0.199878
-0.674631044	pkd Smax/Tmax	-0.206007
-0.680850363	plcgarea	-0.317943
-0.701545492	pkd area	-0.384544

Correlation	Var ID (Primary)	PC2
0.619220029	blnkSmax/Tmax	0.394188
0.227311198	jnk decay rate	0.387993
0.08452165	akt decay rate	0.323753
0.039849629	BLNK area	0.341144
0.035653873	jnk area	0.303462
0.004325512	Blnkdecay rate	0.324988
-0.295971068	baddecay rate	0.0610371
-0.384493909	pkd decay rate	0.0326298
-0.405308952	akt area	0.0117971
-0.624633789	aktSmax/Tmax	-0.334049
-0.645311466	pyk2 Smax/Tmax	-0.263675
-0.674631044	pkd Smax/Tmax	-0.205642
-0.680850363	plcgarea	-0.277888
-0.701545492	pkd area	-0.150393

NFAT

Correlation	Var ID (Primary)	PC1
0.828916901	erk decay rate	-0.0736067
0.37636185	syk decay rate	-0.333904
0.007440069	akt area	-0.409618
-0.014677327	plcg decay rate	-0.458881
-0.390959727	pkd area	-0.484196
-0.400900023	pkd decay rate	-0.457615
-0.656729416	lyn area	0.12836
-0.687734377	bad area	-0.161782
-0.741823004	baddecay rate	-0.19823

Correlation	Var ID (Primary)	PC2
0.828916901	erk decay rate	0.47993
0.37636185	syk decay rate	0.352078
0.007440069	akt area	0.145187
-0.014677327	plcg decay rate	0.183792
-0.390959727	pkd area	-0.0332913
-0.400900023	pkd decay rate	-0.148255
-0.656729416	lyn area	-0.445872
-0.687734377	bad area	-0.445645
-0.741823004	baddecay rate	-0.433679

pp65

Correlation	Var ID (Primary)	PC1
0.509559265	bad Smax/Tmax	0.142951
0.46757556	lyn decay rate	0.185655
0.423286538	lyn area	0.206982
0.313307514	camkiidecay rate	-0.0775227
0.278898409	Camkii area	-0.224048
0.14371287	bcl2 Smax/Tmax	-0.210265
0.124830289	mek area	-0.237577
-0.016870718	syk area	-0.28509
-0.017913145	akt area	-0.229917
-0.055480772	shc decay rate	-0.29133
-0.065386882	bcl2 area	-0.263222
-0.11677101	erk decay rate	-0.153744
-0.155151679	syk decay rate	-0.287704
-0.226593561	shc area	-0.319999
-0.387566295	plcgarea	-0.293228
-0.389077736	pkd Smax/Tmax	-0.246394
-0.413025469	pkd area	-0.280885
-0.454396467	plcg decay rate	-0.172449
-0.56611514	pkd decay rate	-0.194861
-0.92371394	shc Smax/Tmax	-0.0869428

Correlation	Var ID (Primary)	PC2
0.509559265	bad Smax/Tmax	0.201576
0.46757556	lyn decay rate	0.179731
0.423286538	lyn area	0.156412
0.313307514	camkiidecay rate	0.408194
0.278898409	Camkii area	0.38566
0.14371287	bcl2 Smax/Tmax	0.34981
0.124830289	mek area	0.288375
-0.016870718	syk area	0.0894769
-0.017913145	akt area	0.149279
-0.055480772	shc decay rate	0.154678
-0.065386882	bcl2 area	0.266784
-0.11677101	erk decay rate	-0.158455
-0.155151679	syk decay rate	-0.041361
-0.226593561	shc area	0.098189
-0.387566295	plcgarea	0.0373217
-0.389077736	pkd Smax/Tmax	-0.199016
-0.413025469	pkd area	-0.0042797
-0.454396467	plcg decay rate	-0.18169
-0.56611514	pkd decay rate	-0.191965
-0.92371394	shc Smax/Tmax	-0.378636
