# Supplementary material for: Cellular phosphatases facilitate combinatorial processing of receptor-activated signals
Source: BMC Res Notes. 2008 Sep 17;1:81. doi: 10.1186/1756-0500-1-81 (PMC2573882; doi:10.1186/1756-0500-1-81)
Supplement: Additional File 15 — Sensitivity of response specific VIPs to perturbations. Sensitivity of response specific VIPs to perturbations. [file 1756-0500-1-81-S15.pdf]

Additional file 15:Sensitivity of Response specific VIPs to perturbations

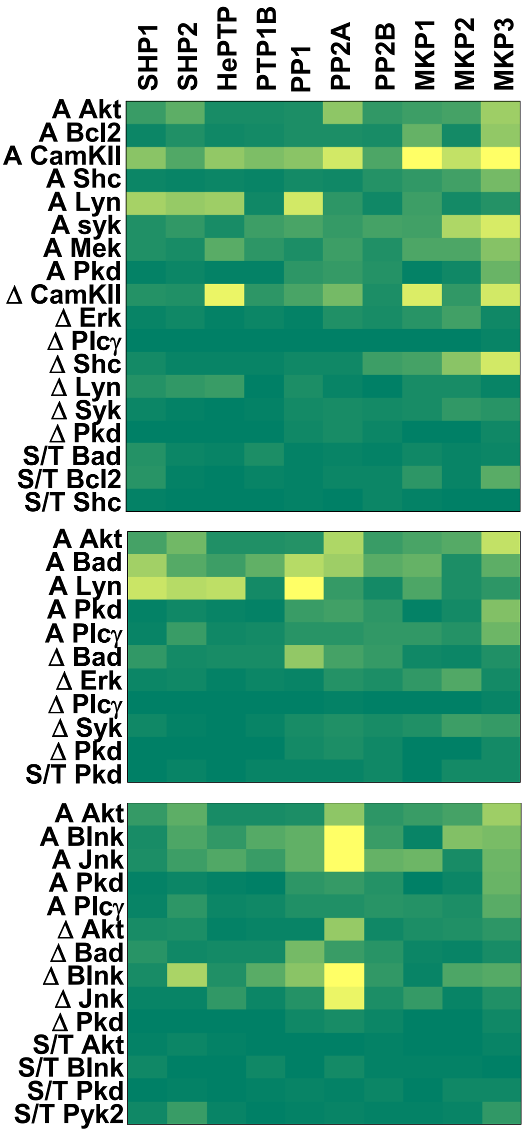

Additional file 15. Sensitivity of response specific VIPs to perturbations.
VIPs from the minimal model for each of the response models were monitored for their sensitivity to the perturbations applied.The sensitivity was calculated as fold variation from the mock siRNA treated condition. From top to bottom, the checkered boxes represent pp65, NFAT and AP1 specific VIPs (mentioned at the left of each row) respectively under all the knockdown conditions (mentioned above every column).
